# Evolutionary Monte Carlo of QM Properties in Chemical Space: Electrolyte Design

**DOI:** 10.1021/acs.jctc.3c00822

**Published:** 2023-11-27

**Authors:** Konstantin Karandashev, Jan Weinreich, Stefan Heinen, Daniel Jose Arismendi Arrieta, Guido Falk von Rudorff, Kersti Hermansson, O. Anatole von Lilienfeld

**Affiliations:** † Faculty of Physics, University of Vienna, Kolingasse 14-16, AT-1090 Wien, Austria; ‡ Vector Institute for Artificial Intelligence, Toronto, M5S 1M1 Ontario, Canada; § Department of Chemistry-Ångström Laboratory, Uppsala University, Box 538, SE-75121 Uppsala, Sweden; ∥ Department of Chemistry, University Kassel, Heinrich-Plett-Str.40, 34132 Kassel, Germany; ⊥ Center for Interdisciplinary Nanostructure Science and Technology (CINSaT), Heinrich-Plett-Straße 40, 34132 Kassel, Germany; # Departments of Chemistry, Materials Science and Engineering, and Physics, University of Toronto, St. George Campus, Toronto, M5S 1A1 Ontario, Canada; ¶ Machine Learning Group, Technische Universität Berlin and Institute for the Foundations of Learning and Data, 10587 Berlin, Germany

## Abstract

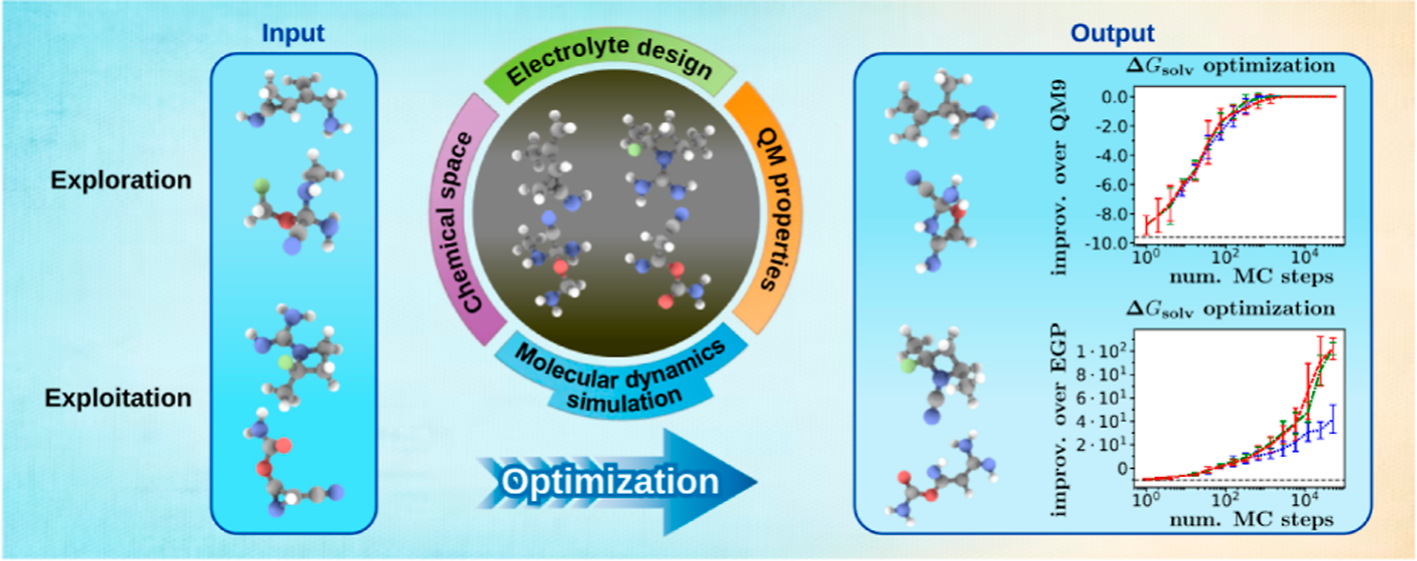

Optimizing a target function over the space of organic molecules is an important problem appearing in many fields of applied science but also a very difficult one due to the vast number of possible molecular systems. We propose an evolutionary Monte Carlo algorithm for solving such problems which is capable of straightforwardly tuning both exploration and exploitation characteristics of an optimization procedure while retaining favorable properties of genetic algorithms. The method, dubbed MOSAiCS (**M**etropolis **O**ptimization by **S**ampling **A**daptively **i**n **C**hemical **S**pace), is tested on problems related to optimizing components of battery electrolytes, namely, minimizing solvation energy in water or maximizing dipole moment while enforcing a lower bound on the HOMO–LUMO gap; optimization was carried out over sets of molecular graphs inspired by QM9 and Electrolyte Genome Project (EGP) data sets. MOSAiCS reliably generated molecular candidates with good target quantity values, which were in most cases better than the ones found in QM9 or EGP. While the optimization results presented in this work sometimes required up to 10^6^ QM calculations and were thus feasible only thanks to computationally efficient *ab initio* approximations of properties of interest, we discuss possible strategies for accelerating MOSAiCS using machine learning approaches.

## 1 Introduction

Increasing efficiency and longevity of energy storage systems is critical for improving economic sustainability of lowering greenhouse gas emissions.^1^ One aspect of this problem is searching chemical compound space for organic molecules optimal for a target application, such as lithium battery electrolyte components^2−6^ or electroactive molecules for redox flow batteries.^7,8^ In this work, we focused on the former, more specifically on finding electrochemically stable organic molecules that are good solvents for alkali salts. While such searches can be aided with high-throughput screening,^2−4^ there has been a surge of ways to go beyond by increasing the efficiency of compound property evaluations, *e.g.*, with machine learning^9^ or quantum alchemy,^10−12^ and by sampling chemical space more efficiently. In the context of optimizing small organic molecules, most methods of the latter category can be classified as those based on Markov decision processes,^13−18^ recurrent neural networks,^19,20^ genetic algorithms,^21−26^ and variational autoencoders.^27,28^

**Figure 1:**
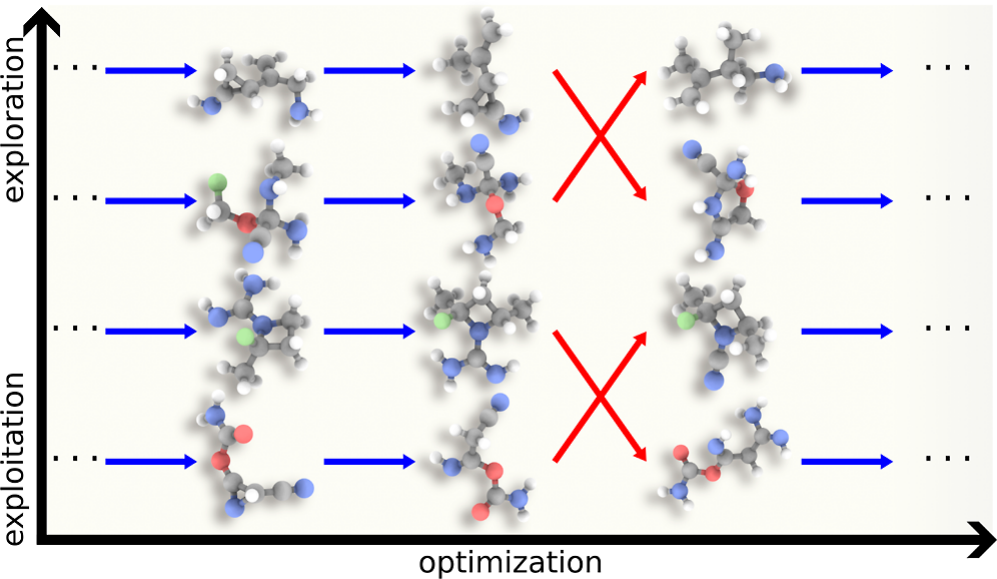
Scheme of an evolutionary Monte Carlo workflow for molecular optimization that introduces several *replicas* with varying degree of focus on *exploitation* or *exploration* aspects of the calculation and evolves them by applying *elementary mutations* (blue) to single replicas and *crossover moves* (red) to pairs of replicas in a way satisfying detailed balance.

While several variants of Markov chain Monte Carlo^29^ sampling have also been applied to molecule optimization problems,^30,31^ one intriguing variant, namely, evolutionary Monte Carlo,^32−34^ has been overlooked so far. The approach combines two philosophies that have demonstrated reliable performance for a range of optimization problems: parallel tempering^35−37^ and genetic algorithms.^38−40^ As illustrated in Figure 1, evolutionary Monte Carlo involves running several Markov chain Monte Carlo simulations that focus on *exploitation* (*i.e.*, refining already known molecules *via* incremental changes) or *exploration* (*i.e.*, finding promising regions of chemical space), which interact by swapping configurations analogously to parallel tempering or by creating “child configurations” as in genetic algorithms in a way that observes detailed balance condition.^41^ As is the case for genetic algorithms, increasing the number of replicas yields more opportunities for creating “child configurations”, thus accelerating exploration of chemical space. However, unlike genetic algorithms, evolutionary Monte Carlo allows straightforward control of its exploration and exploitation aspects while guaranteeing to *eventually* find the global minimum due to the properties of Markov chain Monte Carlo. Evolutionary Monte Carlo can also potentially be combined with nested Monte Carlo techniques^42−44^ to utilize multiple optimized quantity evaluation methods at once, *e.g.*, when laboratory experiments are used alongside theoretical and machine learning approaches,^45−48^ an advantage particularly relevant for high-throughput automated laboratory workflows.^48−51^

With these reasons in mind, we implemented an evolutionary Monte Carlo algorithm inspired by a family of genetic algorithms for optimization in the space of molecular graphs.^21−24^ While some recently proposed methods for molecular optimization operate in string representations,^18,19,25,52,53^ we performed all procedures directly on chemical graphs to facilitate ensuring validity of generated molecules and maintaining detailed balance, as well as provide a more direct connection between the molecules considered and graph-based representations that have proven efficient in machine learning applications.^54,55^ Lastly, we implemented a simple Wang–Landau biasing potential^56^ as a *curiosity reward*(57) increasing exploration aspect of the algorithm by “pushing” our Markov chain Monte Carlo simulations out of previously occupied graphs. The resulting method is named MOSAiCS (**M**etropolis **O**ptimization by **S**ampling **A**daptively **i**n **C**hemical **S**pace). While we were mainly designing our approach with battery applications in mind, we think it should be useful for other molecular optimization problems, such as those arising in drug design.^15,17,19,31,58−60^

The rest of the article is organized as follows. Section 2 first presents the main ideas behind our approach in Subsections 2.1–2.3, following up with a description of the optimization problem on which we test it in Subsection 2.4 and details of our Monte Carlo simulations in Subsection 2.5. Section 3 discusses our experimental results; and Section 4 concludes the paper with a results summary and outline of possible strategies to improve our approach. Some technical details of our method’s implementation, experimental setup, and results are left for the Supporting Information.

## 2 Theory

### 2.1 Chemical Space Definition

**Figure 2:**
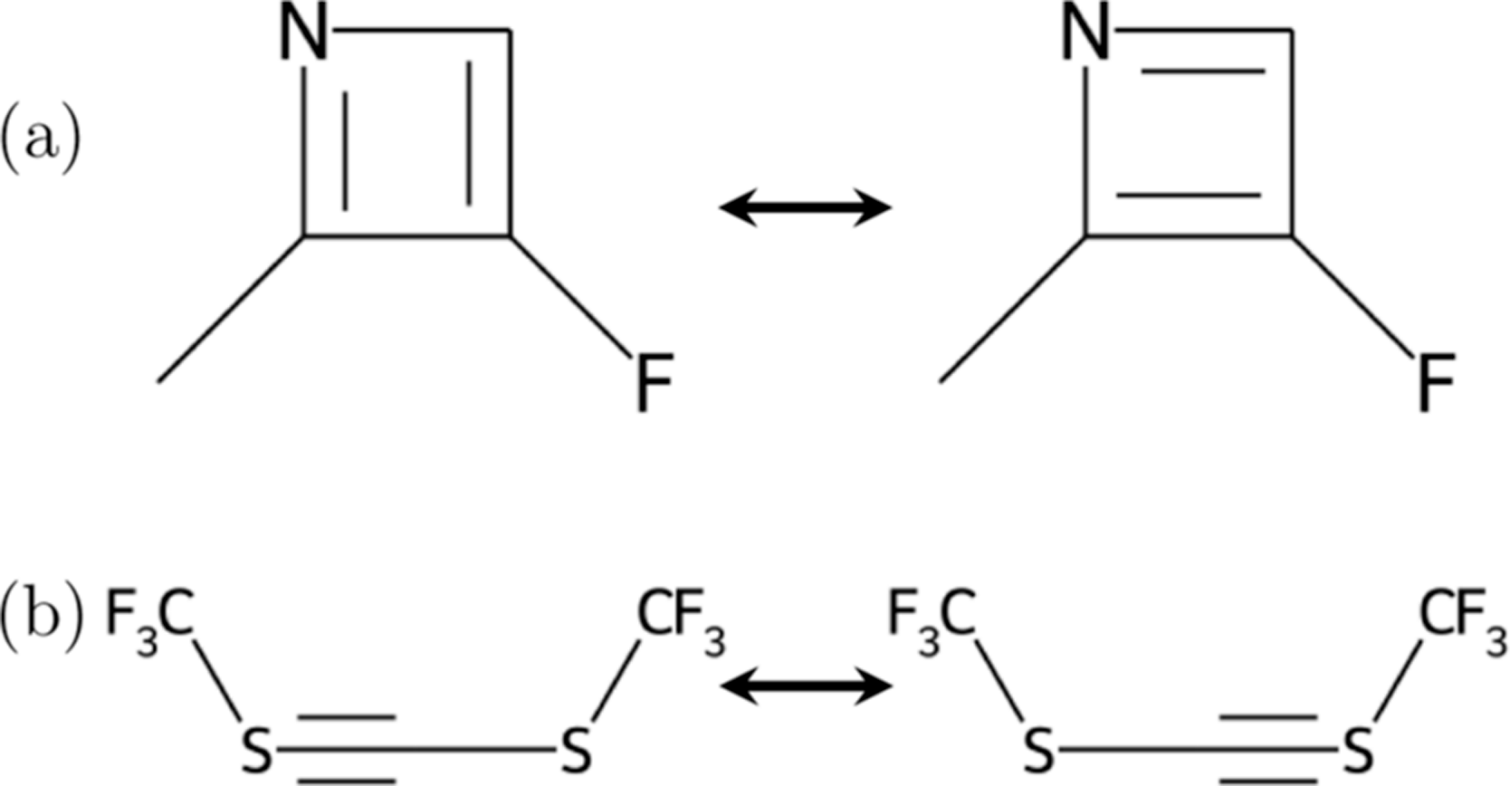
Examples of molecules that can be written in terms of several resonance structures that differ in (a) covalent bond orders and (b) both covalent bond orders and heavy atom valences.

We aim to minimize a loss function *F* over a set of molecules, with the latter represented by their *chemical graphs*. We define a chemical graph as an undirected graph whose *nodes* correspond to heavy atoms, along with, where present, covalently connected hydrogen atoms, and where the *graph edges* connect a pair of nodes if their heavy atoms share a covalent bond. For a chemical graph, we also define a *resonance structure* as a set of *valences* of nodes’ heavy atoms and *orders of covalent bonds* connecting these heavy atoms, both quantities taking integer values. The sum of covalent bond orders connecting a heavy atom to other atoms equals its valence, with the orders of bonds between a heavy atom and a hydrogen atom counted as one. Valence numbers are chosen to be chemically reasonable (*e.g.*, IV for C, II, or IV, or VI for S), and we require their sum to be the minimum needed to build a set of covalent bond orders. We also forbid covalent bond orders larger than three. The reasons for not including valences and bond orders in the definition of a chemical graph, but rather enumerating their possible values separately, are illustrated in Figure 2, demonstrating examples of molecules for which several resonance structures differing in bond orders or bond orders and heavy atom valences can be defined. We emphasize that while our definition of bond orders and valences is loosely based on valence structure theory, it was designed not to reflect actual electronic structure of a molecule but to allow convenient definitions of changes of chemical graphs that are illustrated and discussed in detail further down in Section 2.

Our definition of a chemical graph *a priori* prevents us from differentiating between conformers or stereoisomers, and we will assume our optimization problem to be unaffected by this, *e.g.*, if for a given chemical graph we are interested only in the most stable stereoisomer and we optimize a Boltzmann average. We also did not implement support for molecules where valid Lewis structures can only be generated by assigning charges to atoms, *e.g.*, compounds with nitro groups; hence, they were ignored during all calculations carried out in this work.

### 2.2 Monte Carlo Sampling

We perform optimization by running a Markov chain Monte Carlo simulation (referred to as just “simulation” from now on) of unnormalized probability density similar to the one used for parallel tempering
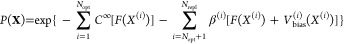
1where **X** is a set of *N*_repl_ chemical graphs (also referred to as *replicas*) *X*^(*i*)^ (*i* = 1, ..., *N*_repl_), β^(*i*)^ (*i* = *N*_opt_ + 1, ..., *N*_repl_) are temperature parameters, *V*_bias_ is the biasing potential, and *C*^∞^ is the “infinitely convex function” defined to be such that for arbitrary sets of numbers *x*^(*j*)^ and *y*^(*j*)^ (*j* = 1, ..., *N*_opt_)
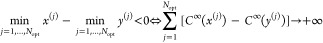
2and *N*_opt_ is the number of replicas that, as will become clear later, effectively undergo greedy stochastic minimization and are referred to as *greedy replicas*, with the other replicas, referred to as *exploration replicas*, providing a less restricted exploration of chemical space and preventing greedy replicas from getting stuck in a local minimum of *F*. The history-dependent biasing potential *V*_bias_^(*i*)^ is defined as^56^
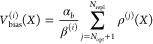
3where ρ^(*j*)^(*X*) is the number of times *X* has been visited during the simulation by replica with index *j* (details on how it was evaluated are left for Subsection 2.5), α_*b*_ is the user-defined bias proportionality coefficient. Setting a nonzero α_*b*_ makes sampling **X** non-Markovian; as a result, our certainty that in this regime a global minimum of *F* w.r.t. *X* is eventually found is based not on properties of Markov chain Monte Carlo but on heuristic expectation that the biasing potential would make probability distribution of each exploration replica approach uniformity, leading to at least one replica finding the global minimum during a finite number of simulation steps.

### 2.3 Monte Carlo Moves

A simulation consists of taking a sequence of *moves* in a way outlined in Algorithm 1. If the current set of replicas is in configuration **X**_1_, a move involves randomly generating parameters **w** of a change and deciding to replace **X**_1_ with the change’s outcome (or *trial configuration*) **X**_2_ with an acceptance probability similar to the standard Metropolis–Hastings expression^41^
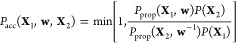
4where *P*_prop_(**X**_1_, **w**) is the probability that **w** is proposed given that **X**_1_ is the initial configuration and **w**^–1^ are parameters of a random change yielding **X**_1_ when applied to **X**_2_ and corresponding to a unique **w**. The latter property ensures that detailed balance still holds in situations when several **w** yield the same trial configuration **X**_2_. Trial configurations decreasing the minimal value of *F* among greedy replicas compared to initial configurations is accepted automatically due to our definition of *C*^∞^ (2).
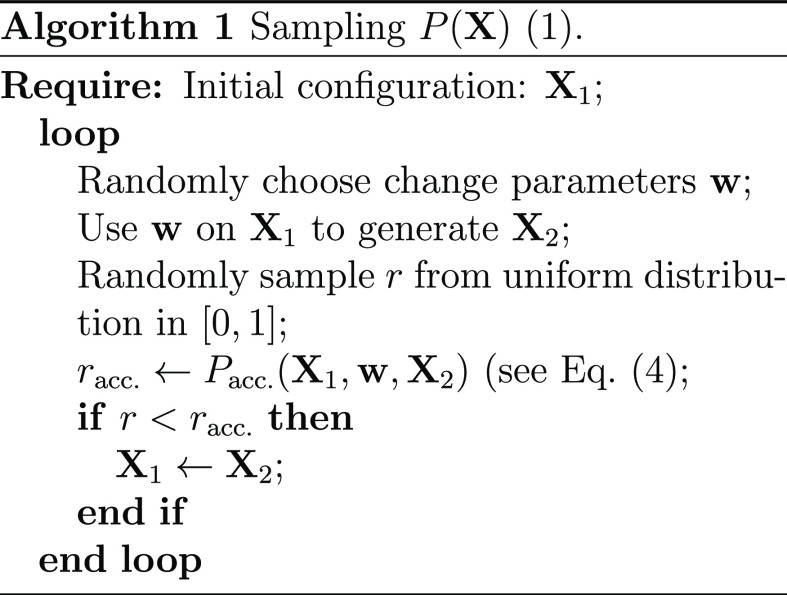


**Figure 3:**
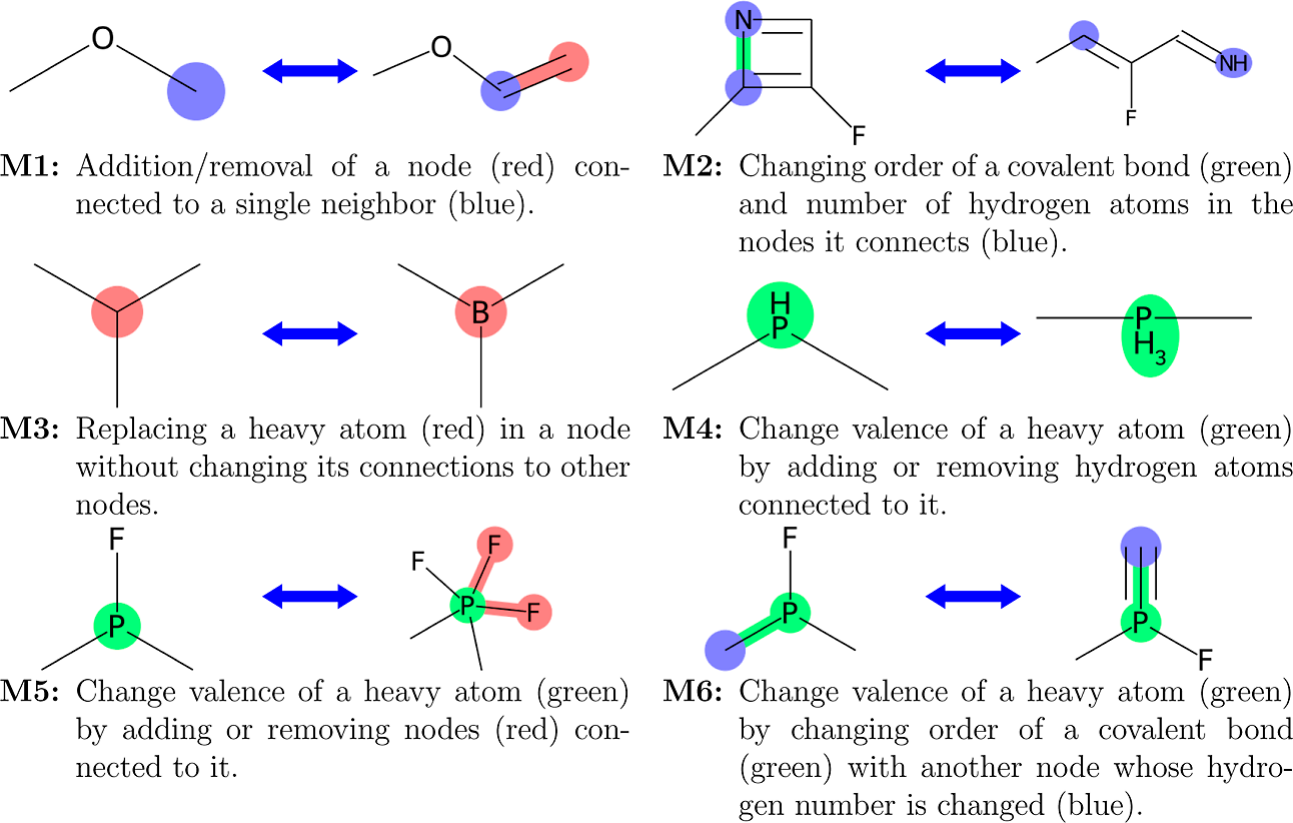
Definitions and examples of elementary mutations. A node is colored green if its heavy atom changes valence; otherwise, it is colored red if it is destroyed or created, or colored blue if it changes the number of hydrogen atoms connected to the heavy atom. A bond is colored red if it is destroyed or created or green if it, in general, changes only bond order, though the latter may involve changing the order to and from 0. The valences in M5 and M6 always change to adjacent values (*e.g.*, for S valence can change from II to IV but not from II to VI); nodes created and destroyed in M1, M3, and M5 contain heavy atoms in their smallest valence state (*e.g.*, valence II for S).

We use three types of moves to propose the trial configurations **X**_2_; we will only discuss the general idea behind them here with implementation details left for Supporting Information. The first type, termed *elementary moves*, applies an *elementary mutation* outlined in Figure 3 to a single replica; such moves correspond to incremental exploration of chemical space. To accelerate greedy optimization of molecules, we additionally introduced the “no reconsiderations condition”: if change parameters **w** corresponding to an elementary move have been rejected for a greedy replica, they are not considered again.

The second type of move is *tempering swap* moves that are analogous to the swap moves in conventional parallel tempering techniques and involve randomly choosing replicas with indices *i* and *j* in such a way that at least one of them is an exploration replica, considering a swap of the corresponding chemical graphs and accepting it with acceptance probability (4). These moves allow greedy replicas stuck in a local minimum of *F* to get to chemical graphs with lower values of *F* if the latter are discovered by an exploration replica.

**Figure 4:**
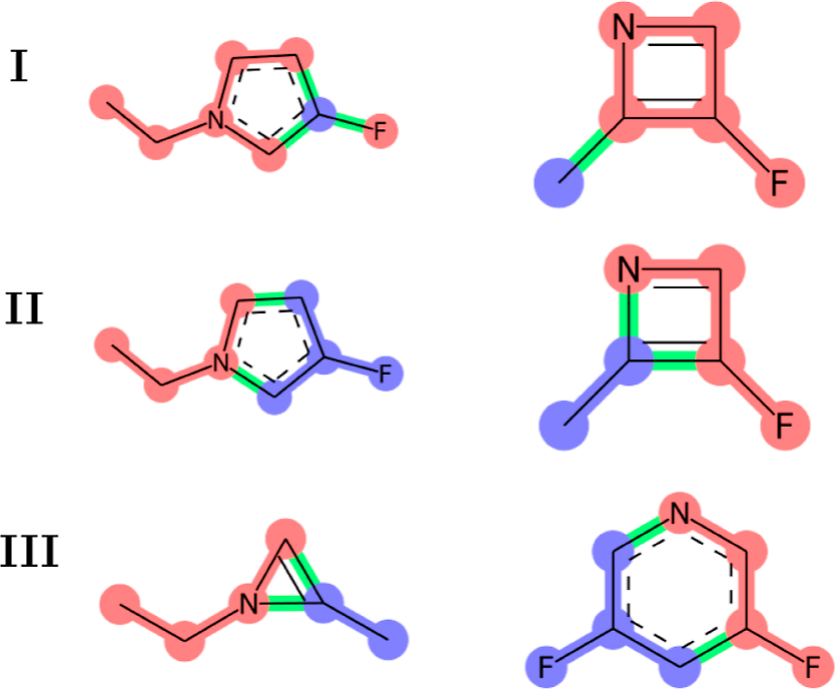
Example of a crossover move. I—selection of two “blue” nodes in two molecules; II—selecting the “blue” neighborhoods of the two blue nodes, coloring the rest “red,” with the bonds connecting blue and red fragments colored “green”; and III—exchanging the blue fragments between molecules and reconnecting them with red fragments by exchanging connected nodes between pairs of green bonds.

The third type of moves are *crossover moves* inspired by the procedure developed in ref (21), which are introduced to allow drastic changes of chemical graphs occupied by replicas. The general idea is illustrated in Figure 4: a pair of nodes is randomly chosen in two chemical graphs, and the neighborhoods of these two nodes are exchanged to create two new chemical graphs. Thus, defined crossover moves are more restrictive than the ones of ref (21) as they do not allow exchanging fragments of arbitrary shape and connectivity. These restrictions, however, make it straightforward to ensure that the resulting chemical graphs satisfy constraints on the number of nodes, are connected, and correspond to a change for which the *P*_prop_(**X**_1_, **w**)/*P*_prop_(**X**_2_, **w**^–1^) ratio in *P*_acc_ (4) can be easily calculated.

Setting *F* to be infinitely large for chemical graphs violating certain user-defined constraints is a general way to enforce the latter on the optimization result. However, it is in general preferable to maintain a given constraint as early as during the proposition of trial configuration **X**_2_ to increase the average acceptance probability and the resulting speed of chemical space exploration. We implemented the corresponding algorithms for maintaining constraints on the number of heavy atoms in a molecule and the kinds of atoms that can share a covalent bond since they are simple to maintain, yet quite important for our applications. Lastly, the question of the moves’ sufficiency to access the chemical space and sets of molecules considered in Section 3 in their entirety is discussed in the Supporting Information.

### 2.4 Minimization Problems

A good battery electrolyte is a good solvent for lithium salts and is electrochemically stable. We approximated the former property with polarity; maximizing a molecule’s polarity was in turn approximated by either maximizing the dipole moment *D* or minimizing (making it more negative) the free energy of solvation in water Δ*G*_solv_. We approximated the electrochemical stability requirement with a lower bound on the HOMO–LUMO Δϵ, with which we approximated the width of the compound’s electrochemical stability window.^2^ While the latter relation is not actually practical for battery design,^61^ we still opted for a Δϵ-based electrochemical stability criterion to connect our work with other compound optimization problems where Δϵ can be used.^62,63^ For both *D* and Δ*G*_solv_ optimization, we constrained the molecules’ Δϵ to be larger than either benzene (*strong* Δϵ *constraint*) or octa-1,3,5,7-tetraene (*weak* Δϵ *constraint*), resulting in four minimization problems of differing difficulty. While in this work we focused on the testing performance of MOSAiCS against these single objective optimization problems, our approach can also be used to optimize several properties at once *via* a suitable multiobjective loss function.^64^

We aimed to estimate Δ*G*_solv_, *D*, and Δϵ as computationally cheaply as possible while being qualitatively correct over a wide range of chemical compounds; the resulting protocol is explained in detail in the Supporting Information. Here, we just mention that for a given chemical graph, we used the MMFF94 force field^65^ to generate molecular conformers, for which we performed GFN2-xTB^66^ calculations with analytical linearized Poisson–Boltzmann model^67^ simulating presence of water. The root-mean-square error (RMSE) that is presented for calculated quantities corresponds to the statistical error from randomness of conformer generation. We used two sets of parameters for our protocol: “converged” that produced reasonable RMSEs for a wide variety of compounds but was relatively computationally expensive and “cheap” that was used during our simulations. From now on, Δ*G*_solv_, *D*, and Δϵ will denote estimates of these quantities obtained with the “converged” protocol, while estimates obtained with the “cheap” protocol will be marked with addition of “cheap” superscript.

Each of the four minimization problems was solved in two sets of molecules based on QM9^68^ and the Electrolyte Genome Project^5^ (EGP) data sets. The QM9 data set consists of 134k molecules containing up to nine heavy atoms (of types C, O, N, and F). We defined the “QM9*” set to consist of molecules (not necessarily in QM9) that also contain up to nine heavy atoms of the same elements as QM9 but are additionally constrained by not allowing bonds between N, O, and F atoms, as well as O–H and H–F bonds, since these covalent bonds are typically associated with increased chemical reactivity. The EGP data set was generated with the Materials Project^69^ workflows in an effort to facilitate discovery of novel battery electrolyte molecules; the version currently hosted on the Materials Project Web site contains 24.5k species in total; neutral species for which MMFF94 coordinates could be generated included 19.7k individual chemical graphs containing up to 92 heavy atoms. These characteristics of the EGP data set were the basis for defining the “EGP*” set, whose molecules (not necessarily in EGP) contain up to 15 heavy atoms (of types B, C, N, O, F, Si, P, S, Cl, and Br, which are elements present in organic molecules of EGP) and, for the sake of chemical stability, do not contain covalent bonds between N, O, F, Cl, and Br, between H and B, O, F, Si, P, S, Cl, or Br, and not S–S or P–P bonds.

We chose 15 as the maximum number of heavy atoms allowed in EGP* molecules because this size restriction is obeyed by 87.0 and 97.0% of EGP’s chemical graphs satisfying weak and strong Δϵ constraints. When choosing which elements cannot share a covalent bond in QM9* and EGP* molecules, we mainly aimed for excluding weak bonds, although we also forbade some relatively strong bonds whose presence can signify molecular reactivity. Since we only consider molecules whose valid Lewis structures can be generated without assigning charges to atoms, H–F and double O–O bonds can only be encountered in hydrogen fluoride and oxygen, which we excluded from consideration due to their corrosive properties. Creating N–N bonds inside an organic compound risks making it prone to releasing nitrogen on excitation, adding functional groups containing double N–O bonds to a molecule risks making the latter prone to self-oxidation, and hydroxyl groups engage relatively easily in reactions involving oxidation or nucleophilic attacks.^70^ We note that in practice managing this kind of reactive behavior would require additional use of more sophisticated compound stability measures.

While both QM9* and EGP* are well-defined and finite sets of chemical graphs, their huge size makes evaluating any of their properties exactly, *i.e.*, through exact enumeration of all their chemical graphs, unfeasible. However, we do summarize properties of intersections of QM9* and QM9, as well as EGP* and EGP, in the Supporting Information.

### 2.5 Simulation Details

During a simulation, we used Δϵ^cheap^ to estimate whether a molecule satisfies the constraint on Δϵ; dimensionless loss functions corresponding to *D* and Δ*G*_solv_ were defined as
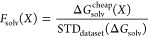
5
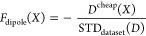
6where STD_dataset_ refers to standard deviation of a quantity over molecules at the intersection between the chemical graph set of interest and the reference data set (QM9 for QM9* and EGP for EGP*) which satisfy the Δϵ constraint of interest. We chose 1000 “pre-final” molecules exhibiting the smallest value of loss function out of the molecules visited during the simulation and evaluated converged estimates of the quantities of interest for them; the molecule with the best *D* or Δ*G*_solv_ value among prefinal molecules satisfying the Δϵ constraint is the one considered the *candidate* molecule proposed by the simulation.

We used *N*_repl_ = 36 with *N*_opt_ = 4 (*cf.* definitions in Subsection 2.2); virtual temperature parameters β^(*i*)^ appearing in *P* (1) were defined in such a way that the smallest and largest β^(*i*)^ were 1 and 8, and the other β^(*i*)^ formed a geometric progression between the two extrema values, the latter being a simple recipe taken from applications of parallel tempering to configuration space sampling.^71,72^ A simulation consisted of 50,000 “global” steps, out of which 60% were “simple” steps applying an elementary move to each replica, 20% were “tempering” steps making tempering swap moves on 128 randomly chosen pairs of replicas, and another 20% were “crossover” steps making crossover moves on 32 randomly chosen pairs of replicas. ρ^(*j*)^(*X*) appearing in *V*_bias_^(*i*)^ (3) was counted as the number of times that replica *j* was found in *X* after a global step had been completed. For elementary moves, we additionally set that the nodes added or removed during M1 mutation could be connected to the molecule with bonds of order from 1 to 3; bonds changed with M2 and M6 mutations could have their order increased or decreased by 1 and 2, respectively; and nodes added or removed with M5 mutation could be connected to the molecule with bonds of order 1 or 2.

We set α_*b*_ to 0.0, 0.2, or 0.4; for each of the resulting 12 combinations of α_*b*_ and optimization problem, we ran eight simulations with different random number generator seeds. For all simulations, all replicas initially occupied the chemical graph of methane. While it would be natural to assign each replica a randomly chosen molecule from the intersection of QM9 and QM9* or EGP and EGP*, we went with the intentional handicap of using methane as the starting molecule to demonstrate that MOSAiCS is capable of constructing all the candidate molecules presented in this section from scratch. The effect of choice of initial conditions on the final result is briefly addressed in the Supporting Information.

## 3 Results and Discussion

In this section, we describe the main results of our numerical experiments. The more technical aspects, such as full information on generated candidates and influence of biasing potential on the search efficiency, are left for Supporting Information.

**Figure 5:**
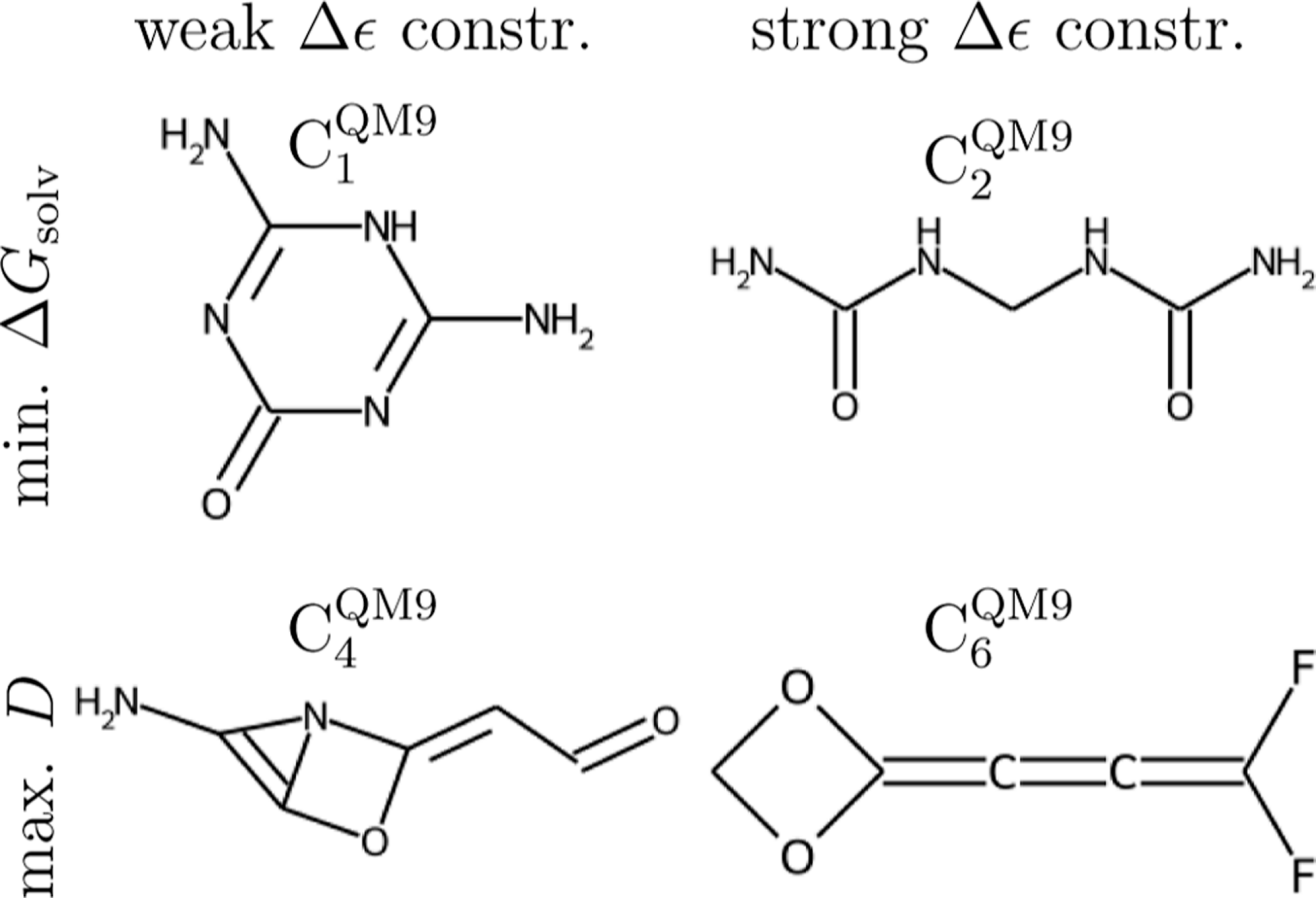
QM9* candidates that exhibited the smallest free energy of solvation Δ*G*_solv_ or largest dipole *D* under weak or strong constraint on the HOMO–LUMO gap Δϵ. See the Supporting Information for more information about them.

**Figure 6:**
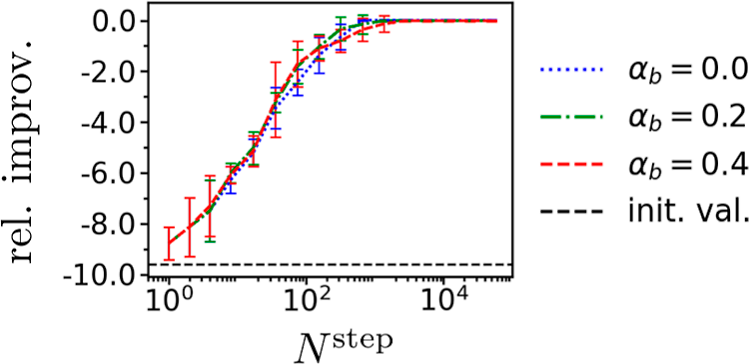
Relative improvement (as defined in Section 3 and estimated from Δ*G*_solv_^cheap^) observed at *N*^step^ global Monte Carlo steps for QM9* simulations optimizing Δ*G*_solv_ with weak Δϵ constraint. For each bias proportionality coefficient α_*b*_, we plot the mean over different random generator seeds, and the error bars correspond to standard deviation. “init. val.” is the relative improvement of the simulations’ starting molecule, *i.e.*, methane.

**Figure 7:**
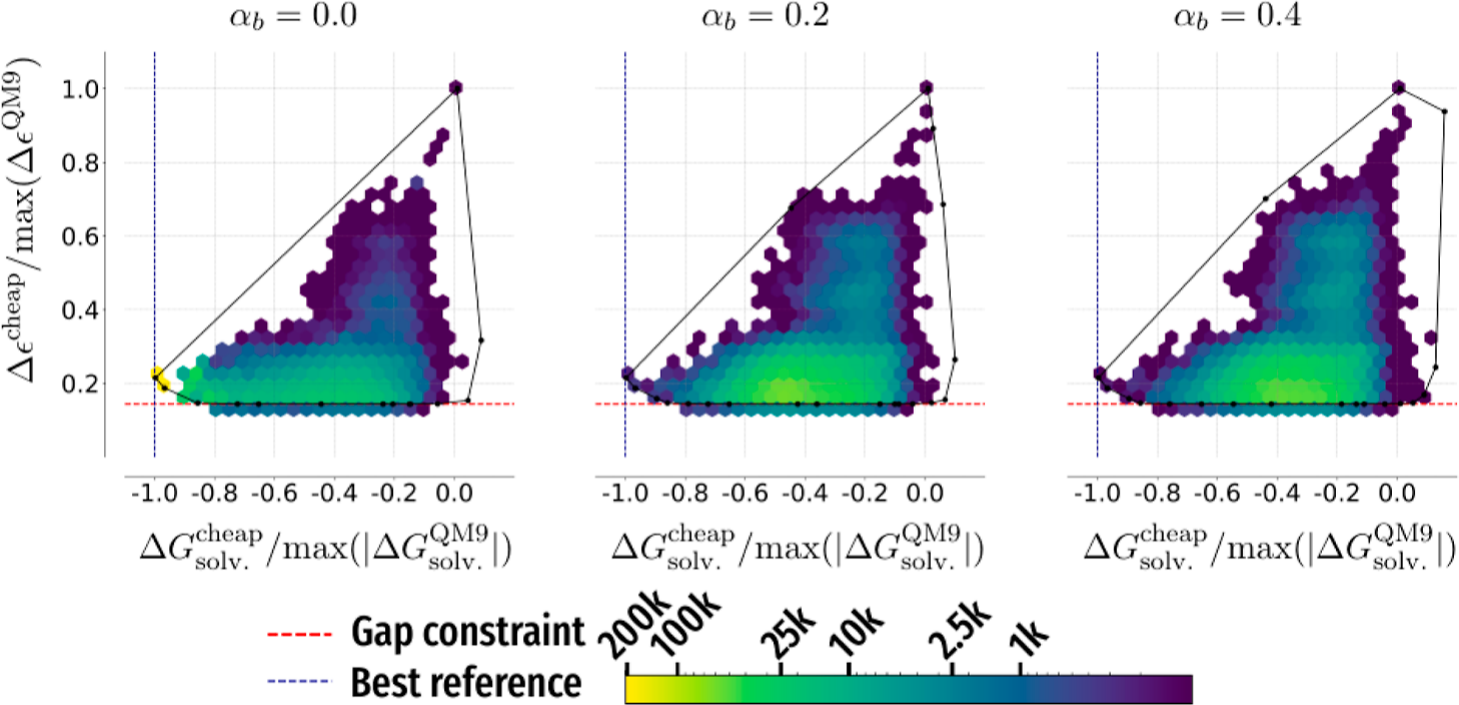
Densities of molecules encountered by example simulations minimizing the free energy of solvation Δ*G*_solv_ with weak HOMO–LUMO gap Δϵ constraint in QM9* for different values of bias proportionality coefficient α_*b*_.

While we ran in total 96 simulations in QM9*, or 24 simulations with different random generator seed and α_*b*_ values for each optimization problem, they agreed remarkably often on candidates proposed, yielding only 10 candidates in total. Table 1 summarizes the best and worst values of optimized quantities for the candidates proposed by MOSAiCS along with the corresponding relative improvement, which we define as absolute difference between a candidate’s optimized quantity value and the corresponding value for the best molecule for the optimization problem taken from the reference data set (*cf.*Table S2 in Supporting Information), divided by the corresponding STD_dataset_. For optimization of Δ*G*_solv_ with weak Δϵ constraint, all trajectories proposed the minimum of Δ*G*_solv_ already present in QM9, while for all other optimization problems, all trajectories proposed candidates that improved significantly on molecules in QM9. The best candidates proposed for a given optimization problem are shown in Figure 5; note that to facilitate discussion of candidates’ properties in Supporting Information, each candidate is referred by a capital *C* with a unique index subscript and a superscript denoting the reference data set. We see how MOSAiCS successfully constructed complex conjugated bond structures facilitating charge transfer, which, in turn, led to smaller, *i.e.*, more negative, Δ*G*_solv_ or larger *D*. Figure 6 displays the optimization progress against the number of global Monte Carlo steps for different α_*b*_ values for minimizing Δ*G*_solv_ with weak Δϵ constraint. We observe convergence of the optimized property with a rate not significantly affected by changing α_*b*_; the same is true with varying degree for other optimization problems as discussed in Supporting Information. To visualize how simulations explored chemical space for different optimization problems and values of α_*b*_, for each such combination we took a simulation that had produced the best candidate and plotted the density of molecules it encountered with respect to the optimized quantity and Δϵ. Figure 7 presents such plots for minimizing Δ*G*_solv_ with the weak Δϵ constraint; plots for other optimization problems are presented and discussed in Supporting Information. We see that increasing α_*b*_ tended to increase the diversity of molecules encountered during the simulations, but this was mainly achieved by considering more molecules in regions of chemical space with larger values of Δ*G*_solv_.

**Table 1:** Best and Worst QM9* Candidates Proposed during Minimization of Free Energy of Solvation Δ*G*_solv_ or Maximization of Dipole *D* with Weak or Strong Constraint on the HOMO–LUMO Gap Δϵ, along with Their Optimized Quantity Values and the Relative Improvement Compared to QM9 Data Set as Defined in Section 3^a^

optimized quantity	Δϵ constraint	optimized quantity value		relative improvement	
		best	worst	best	worst
Δ*G*_solv_	weak	–94.79 ± 0.06		0.004 ± 0.009	
	strong	–68.19 ± 0.60	–67.27 ± 0.00	1.665 ± 0.099	1.537 ± 0.052
*D*	weak	15.73 ± 0.10	15.23 ± 0.00	1.287 ± 0.052	1.013 ± 0.000
	strong	11.14 ± 0.00	10.00 ± 0.00	1.924 ± 0.030	1.087 ± 0.030

a Δ*G*_solv_ and *D* values are given in kJ/mol and debye. The full list of candidate molecules can be found in the Supporting Information.

**Figure 8:**
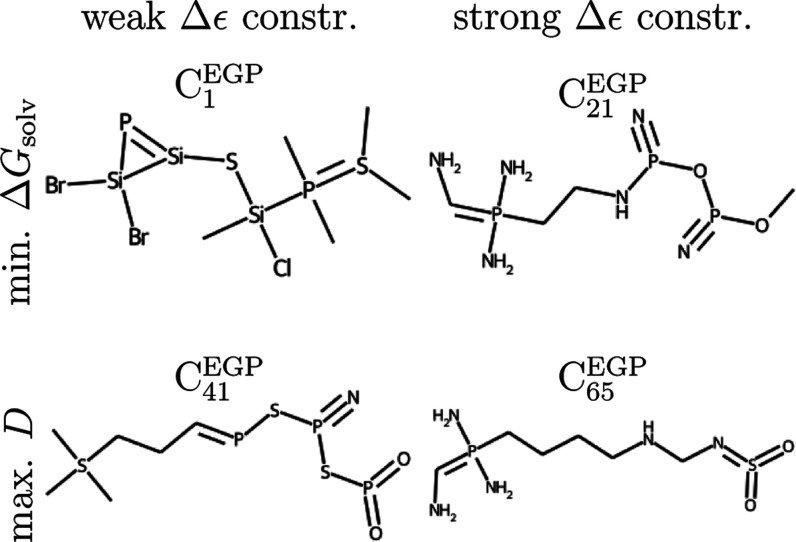
EGP* candidates that exhibited the smallest free energy of solvation Δ*G*_solv_ or largest dipole *D* under weak or strong constraint on the HOMO–LUMO gap Δϵ. See the Supporting Information for more information about them.

**Figure 9:**
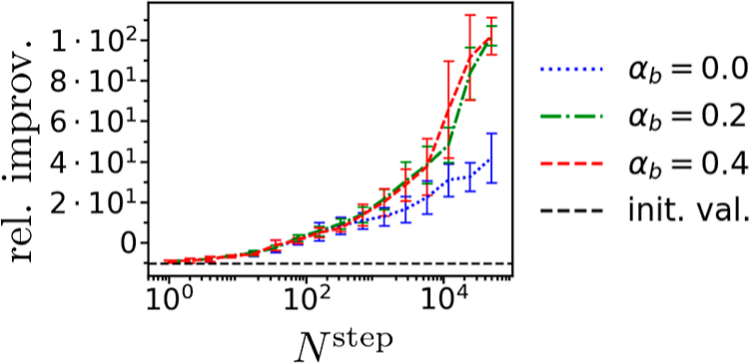
Relative improvement observed at *N*^step^ global Monte Carlo steps for EGP* simulations optimizing Δ*G*_solv_ with the weak Δϵ constraint; results are organized analogously to Figure 6.

**Figure 10:**
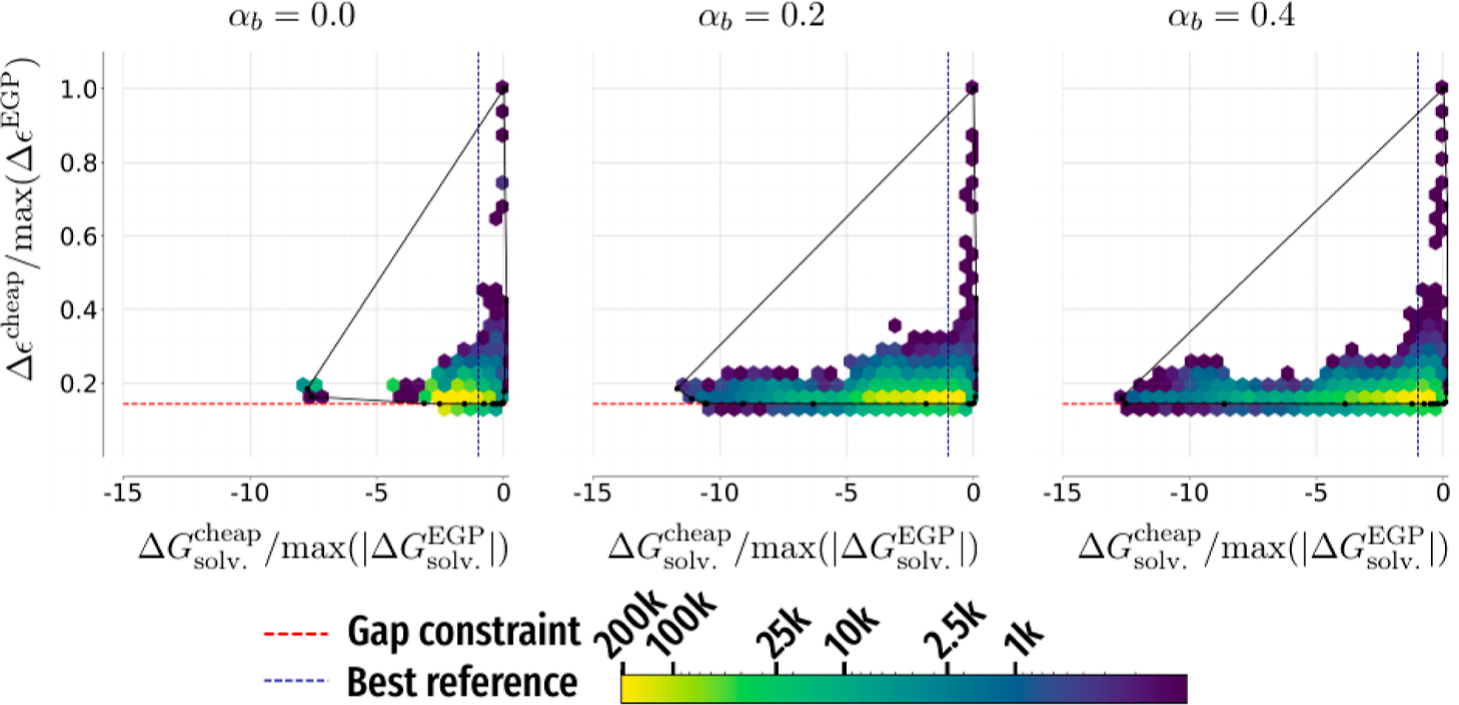
Densities of molecules encountered by simulations minimizing free energy of solvation Δ*G*_solv_ with weak HOMO–LUMO gap Δϵ constraint in EGP* that produced the candidate with the smallest Δ*G*_solv_ for a given value of bias proportionality coefficient α_*b*_.

Optimization in EGP* was harder than that in QM9* due to the larger size of the former set of molecules, resulting in our protocol generating underconverged simulations that rarely agreed on candidates, producing 83 candidates in total. However, as summarized in Table 2, we still observed significant improvement of optimized quantities compared to EGP, although the improvements’ impressive magnitudes are largely due to EGP containing a much less representative portion of EGP* compared to the case of QM9 and QM9*. Best EGP* candidates for each optimization problem are presented in Figure 8; unlike QM9* candidates, no chemical intuition is seen in how they were constructed beyond adding as many polar covalent bonds as possible, which may be due to the underconvergence of our EGP* simulations. The underconvergence can also be observed in our optimization progress plots, the plot for minimizing Δ*G*_solv_ with weak Δϵ constraint presented in Figure 9 and the rest found in the Supporting Information. Figure 9 also demonstrates how adding α_*b*_ can accelerate optimization as a function of global Monte Carlo steps, although we need to note that simulations with larger α_*b*_ on average process more chemical graphs per global Monte Carlo steps, as discussed in Supporting Information. Densities of molecules encountered during simulations minimizing Δ*G*_solv_ with weak Δϵ constraint that produced the best candidates are presented in Figure 10; unlike the case of QM9*, increasing α_*b*_ helped simulations explore parts of the chemical space with smaller values of Δ*G*_solv_. Analogous plots for other optimization problems in EGP* are presented in the Supporting Information.

**Table 2:** Best and Worst EGP* Candidates, Data Notation Is Analogous to Table 1^a^

optimized quantity	Δϵ constraint	optimized quantity value		relative improvement	
		best	worst	best	worst
Δ*G*_solv_	weak	–1194 ± 7	–382.2 ± 1.2	118.0 ± 0.7	30.84 ± 0.13
	strong	–269.8 ± 2.1	–207.6 ± 0.9	23.19 ± 0.25	15.65 ± 0.11
*D*	weak	109.5 ± 0.9	53.69 ± 0.12	51.16 ± 0.48	21.49 ± 0.07
	strong	59.81 ± 1.14	25.88 ± 0.17	34.70 ± 0.76	11.95 ± 0.12

a Full list of candidate molecules can be found in the Supporting Information.

## 4 Conclusions and Outlook

We have proposed an effective algorithm for optimization in chemical space, dubbed MOSAiCS, and successfully applied it to several test optimization problems connected to the lithium battery electrolyte design. In the current implementation, it is only feasible to optimize estimates of quantities of interest that can be evaluated with little computational effort due to the large number of evaluations of loss function made during a simulation (see the Supporting Information); given successes in using active learning for optimization problems in both configuration^73−76^ and chemical^58,77,78^ space, our first priority is to combine MOSAiCS with a similar protocol to decrease the number of loss function evaluations carried out during the simulations. Successful use of Markov Decision Process formalism to accelerate genetic algorithms in chemical space^59^ suggests that MOSAiCS might similarly be improved with a smarter policy for choosing elementary mutations and crossover moves. On a more general note, any method for generating chemical graphs that can also provide corresponding proposition probability *P*_prop_ needed for *P*_acc_ (4) can be integrated into the MOSAiCS framework directly.

While we aimed to propose an approach that would be agnostic to how much is known about the chemical graph set of interest, we still relied on QM9 and EGP to get reasonably rescaled loss functions *F*_solv._ (5) and *F*_dipole_ (6) that were then used during simulations in QM9* and EGP*. This dependence on previously published data should become avoidable by implementing more sophisticated schemes^79^ for adjusting temperature parameters β^(*i*)^ based on trajectory history. Also, while we used heavy atoms with connected hydrogens as nodes of chemical graphs to maximize the chemical diversity of generated compounds, it is possible to expand the algorithm to using larger compound fragments as nodes instead. If applicable to the optimization problem at hand, this modification should simplify the search by both decreasing effective dimensionality of the graphs considered^26,31^ and improving scalability of machine learning models for the molecules of interest.^80,81^

## Supporting Information Available

Details of MOSAiCS implementation and quantity of interest evaluation, discussion of accessibility of QM9* and EGP* by our simulations, and additional experimental results (PDF)

## Notes

Code Availability: Python implementation of MOSAiCS is available at https://github.com/chemspacelab/mosaics.
