# Supplementary material for: Evolutionary Monte Carlo of QM Properties in Chemical Space: Electrolyte Design
Source: J Chem Theory Comput. 2023 Nov 27;19(23):8861–70. doi: 10.1021/acs.jctc.3c00822 (PMC10720348; doi:10.1021/acs.jctc.3c00822)
Supplement: Supplementary file 1 — ct3c00822_si_001.pdf [file ct3c00822_si_001.pdf]

Supporting Information: Evolutionary Monte Carlo of QM properties in chemical space: Electrolyte design

Konstantin Karandashev,[*,†] Jan Weinreich,[†] Stefan Heinen,[‡] Daniel Jose Arismendi Arrieta,[¶] Guido Falk von Rudorff,[§,‖] Kersti Hermansson,[¶] and O. Anatole von Lilienfeld[‡,⊥,#]

†*University of Vienna, Faculty of Physics, Kolingasse 14-16, AT-1090 Wien, Austria*

‡*Vector Institute for Artificial Intelligence, Toronto, ON, M5S 1M1, Canada*

¶*Department of Chemistry-Ångström Laboratory, Uppsala University, Box 538, SE-75121 Uppsala, Sweden*

§*University Kassel, Department of Chemistry, Heinrich-Plett-Str.40, 34132 Kassel, Germany*

‖*Center for Interdisciplinary Nanostructure Science and Technology (CINSaT), Heinrich-Plett-Straße 40, 34132 Kassel*

⊥*Departments of Chemistry, Materials Science and Engineering, and Physics, University of Toronto, St. George Campus, Toronto, ON, Canada*

#*Machine Learning Group, Technische Universität Berlin and Institute for the Foundations of Learning and Data, 10587 Berlin, Germany*

E-mail: konstantin.karandashev@univie.ac.at

1 Details of elementary mutation and crossover move implementations

While in Subsec. 2.3 of the main text it was convenient to speak about invertible elementary mutations, the corresponding procedures were implemented in terms of *elementary changes* listed in the left column of Table S1, which differ from elementary mutations in treating procedures for creating and destroying nodes separately rather than part of unified mutations changing the number of nodes in a chemical graph. To apply a random mutation to a chemical graph we enumerate all sets of possible elementary change parameters, which is also when we observe constraints on allowed types of covalent bonds and number of nodes. We then randomly choose change parameters among those enumerated, by first choosing the elementary change, and then all other parameters in the order listed in the right column of Table S1. Lastly, we check that straightforward application of the change yields a molecule without nodes with invalid valences and if that is not the case reject the move automatically. The last step's necessity is illustrated with an example in Figure S1.

In Table S1, whenever we mention choosing a node or a pair of nodes we mean choosing them among a list of all nodes or pairs of nodes such that none of them are equivalent to any other in the chemical graph. This prevents MOSAiCS from considering several mutations that yield the same chemical graph X_2 given an initial chemical graph X_1, making it straightforward to define the unique inverse change parameters that yield X_1 when applied to X_2. Enumerating all possible changes for X_2 analogously to X_1 allows straightforward calculation of the proposition probability ratio in acceptance probability expression [Eq. (4) in the main text]. Choosing resonance structure for E2 and E6 is necessary in situations when decreasing bond order, depending on the resonance structure, can yield two distinct molecules corresponding to cases when the changed covalent bond is conserved or broken; such situations are illustrated in Figure S2.

Choosing the parameters of a crossover move starts with assigning the "blue fragments"

Table S1: Elementary changes used in this work along with the corresponding order in which random change parameters were chosen.

Elementary change	Choice order
add node (E1a)	1. added node's heavy atom element<br>2. node to which the new node will be connected with a covalent bond<br>3. order of the new covalent bond
remove node (E1b)	1. removed node's heavy atom element<br>2. removed node
change bond order (E2)	1. step by which bond order is changed<br>2. altered pair of nodes<br>3. resonance structure
replace heavy atom (E3)	1. node's new heavy atom element<br>2. changed node
change valence / change hydrogen number (E4)	1. node whose valence is changed<br>2. new number of hydrogens connected to the node
change valence / add heavy atoms (E5a)	1. created nodes' heavy atom element<br>2. node to which the created nodes will be connected via covalent bonds<br>3. order of the new covalent bonds (also automatically defines the number of added nodes)
change valence / remove heavy atoms (E5b)	1. removed nodes' heavy atom element<br>2. node whose neighbors will be removed<br>3. order of covalent bonds connecting removed nodes to the molecule (also automatically defines the number of removed nodes)
change valence / change bond order (E6)	1. step by which bond order is changed<br>2. altered pair of nodes<br>3. resonance structure

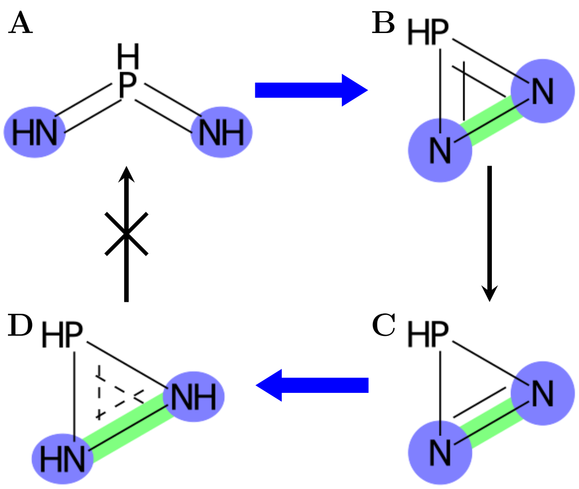

Figure S1: Example of an elementary change that is irreversible due to our definition of valid valence of an atom. Applying elementary change E2 to chemical graph **A** yields chemical graph **B**, which is actually an incorrectly written chemical graph **C**. Applying the inverse of **A**→**B** to **C** yields **D**, which differs from **A**.

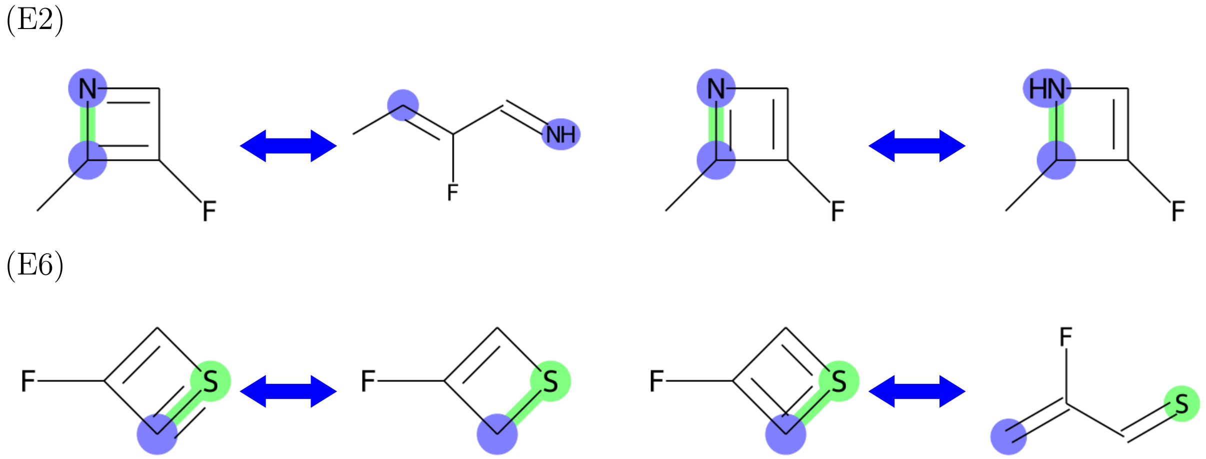

Figure S2: Examples of E2 and E6 elementary changes that decrease order of the covalent bond between the same pairs of nodes by the same values, but yield different results depending on the resonance structure in which the molecules are initialized.

(as illustrated in Figure 4) which are defined as the set of nodes separated by a given number of covalent bonds from an "origin" node. Origin nodes in the two molecules are chosen randomly; then we enumerate all possible blue fragments such that:

- a pair of resonance structures chosen for both molecules allows a one-on-one mapping between green (see Figure 4) covalent bonds of the same order;
- exchanging the blue fragments yields chemical graphs satisfying constraints on the number of nodes in a chemical graph;
- at least one blue fragment and at least one red (see Figure 4) fragment contain more than one node, ensuring that the crossover move is not a simple combination of two E3 elementary changes;
- the number of green bonds is not larger than a certain threshold (set 3 in this work); this is done to limit the number of chemical graph pairs enumerated during the next step.

With the blue fragments chosen we enumerate all pairs of chemical graphs obtained by exchanging them, *i.e.* taking pairs of green bonds from molecules and exchanging nodes between them, thus connecting each molecule's blue fragment to the red fragment of the other molecule. For each thus generated pair we additionally check that the valences in the exchanged blue and red fragments are valid in the new molecules (to prevent situations analogous to the one illustrated in Figure S1), that none of the newly created green bonds connect the same pair of nodes (to prevent irreversible changes illustrated in Figure S3), and that none of the newly created bonds violate constraints on which types of heavy atoms can be covalently connected.

Of the resulting pairs of chemical graphs one pair, consisting of chemical graphs $\tilde{X}'$ and $\tilde{X}''$, is randomly chosen. The chemical graphs are assigned to replicas i' and i'' according to the value of sampled probability P [Eq. (1) in the main text] corresponding to the ordering.

In other words, if both replicas i' and i'' are greedy the assignment is random; if exactly one is greedy it is assigned the chemical graph corresponding to the smaller value of F; if both are exploration replicas the probability of assigning $\tilde{X}'$ and $\tilde{X}''$ to replicas i' and i'' is proportional to

$$P_{\text{order}}(\tilde{X}', \tilde{X}'', i', i'') = \exp\left\{-\beta^{(i')}\left[F(\tilde{X}') + V_{\text{bias}}^{(i')}(\tilde{X}')\right] - \beta^{(i'')}\left[F(\tilde{X}'') + V_{\text{bias}}^{(i'')}(\tilde{X}'')\right]\right\}. \quad (1)$$

Note that if i' and i'' are a greedy and an exploration replicas and $F(X^{(i')}) > F(X^{(i'')})$ then the crossover move has no inverse; we avoided such situations by preceding and following a crossover move with attempting a tempering swap move on replicas i' and i''. The inverse crossover parameters required to calculate acceptance probability P_{acc} [Eq. (4) in the main text] correspond to the same blue fragments being exchanged.

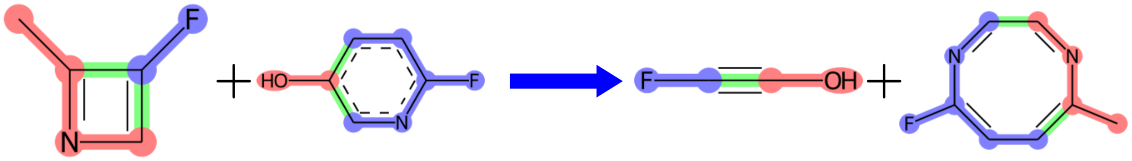

Figure S3: Example of how not checking conservation of number of green bonds during a crossover move results in a non-invertible procedure.

2 Experimental details

2.1 Estimating quantities of interest

To estimate free energy of solvation ΔG_{solv}, dipole D, and HOMO-LUMO gap $\Delta\epsilon$ we used the recipe from Ref. S1, as implemented in the Morfeus package,[S2] to generate the molecule's conformers and Boltzmann weights $w_B^{(k)}$ (where k is conformer index) at $T = 298.15\,\text{K}$ with the MMFF94 forcefield[S3] as implemented[S4] in RDKit.[S5] If converting the lower energy conformer's geometry back to a chemical graph using the xyz2mol code[S6,S7] proved impossible or yielded a chemical graph different from the initial one we considered conformer generation

to have failed. To decrease the number of conformers considered, we introduced "cut" Boltzmann weights $\tilde{w}_B^{(k)}$ obtained by solving w.r.t. $\tilde{w}_B^{(k)}$ and ρ_-

$$\forall k : \tilde{w}_B^{(k)} = \max\left(w_B^{(k)} - \rho_-, 0\right), \tag{2}$$

$$\frac{\sum_k \tilde{w}_B^{(k)}}{\sum_k w_B^{(k)}} = 1 - \rho_{\text{cut}}, \tag{3}$$

where ρ_{cut} is a user-defined parameter. Note that $\tilde{w}_B^{(k)}$ is exactly zero for higher-energy conformers of the molecule while being a smooth function of $w_B^{(k)}$. Then for each conformer with non-zero $\tilde{w}_B^{(k)}$ we used xtb code[S8] to run GFN2-xTB[S9] calculations with analytical linearized Poisson-Boltzmann (ALPB) model[S10] used to simulate presence of water. For D and $\Delta\epsilon$ we took weighted averages of the resulting dipole and HOMO-LUMO gap estimates with $\tilde{w}_B^{(k)}$ as weights; for ΔG_{solv} the averaged quantity was the difference between GFN2-xTB electronic energies obtained with and without ALPB. Since there is a randomness to generating conformers with Morfeus, the procedure was repeated N_{repeat} times with the mean taken as final result and the standard deviation used to estimate the latter's root mean square error (RMSE). A molecule was considered invalid if one of the N_{repeat} generations of MMFF94 coordinates failed or if one of the GFN2-xTB calculations done for the molecule did not converge. This *a priori* limited us to molecules that are not geometrically strained, but we did not consider this restriction important for our end goal applications since geometrically strained molecules are in general less thermodynamically stable, making them less desirable as battery electrolyte components. Lastly, we had to restart some EGP* simulations due to our code's failure to handle some xtb code exceptions. Since we encountered such exceptions during less than one in 10^7 of evaluation of EGP* molecules this issue had a negligible impact on the results presented in this work.

The two different sets of calculation parameters mentioned in Subsec. 2.4 of the main text are $N_{\text{conf}} = 32$ and $N_{\text{repeat}} = 16$ for "converged," with $N_{\text{conf}} = 8$ and $N_{\text{repeat}} = 1$ for "cheap"; $\rho_{\text{cut}} = 0.1$ was chosen for both cases. Quantity estimates obtained with the former

exhibited low RMSE's over QM9 and EGP datasets, but sometimes required relatively large computational time to be calculated, hence the cheap set of parameters were used during our simulations.

Properties of ΔG_{solv}, D, and $\Delta\epsilon$ over molecules in intersections of QM9 with QM9* and EGP with EGP* satisfying $\Delta\epsilon$ constraints are summarized in Table S2. Note that RMSE's of properties of interest are reasonable over all such molecules. The table also presents reference values used to calculate relative improvements presented in the main text for candidate molecules.

2.2 Accessibility of chemical space

To guarantee (at least for $\alpha_b = 0$ when the sampling is Markovian) that MOSAiCS eventually finds the minimum of loss function over the set of chemical graphs of interest the procedures used to propose trial replica configurations should have *connectivity*, *i.e.* it should be possible to morph each pair of members of the set from one into another using these procedures alone and with only other molecules in this set as intermediates. Mutations M1-M4 are enough to guarantee connectivity in a large variety of relevant sets of chemical graphs, for example those obeying an upper bound on the number of heavy atoms and a constraint on which types of non-carbon heavy atoms can share a covalent bond. Connectivity of this kind of sets is easily seen by "constructing" and "deconstructing" each set member to and from methane. The same argument holds for QM9* but, unfortunately, not for EGP* due to P and S atoms being forbidden to share a covalent bond with a hydrogen atom, making it impossible to use mutation M4. Introducing additional mutations M5 and M6 alleviates the problem only partially, with an example of an EGP* molecule that cannot be transformed to and from methane using elementary mutations presented in Figure S4. This molecule can be considered by the simulations used in this work thanks to crossover moves, but we cannot guarantee this is the case for all such examples. However, we note that all such molecules are geometrically strained, which makes them not interesting for our applications due to

Table S2: Properties of sets of molecules at the intersection of QM9 and QM9* or EGP and EGP* that satisfy weak or strong constraint on HOMO-LUMO gap $\Delta\epsilon$, namely their number (num. mol.), standard deviations (STD) of optimized quantities (free energy of solvation ΔG_{solv} and dipole D) over these molecules, the maximum root mean square errors (RMSEs) observed ΔG_{solv}, D, and $\Delta\epsilon$, minimum of ΔG_{solv} and maximum of D, along with SMILES of the molecules for which these extrema values were observed. The total number of molecules (tot. num. mol.) is the number of molecules whose chemical graphs, if extractable from QM9 or EGP coordinates with xyz2mol code, satisfied constraints of QM9* or EGP* sets.

$\Delta\epsilon$ constr.	weak	strong
	QM9 and QM9* intersection	
tot. num. mol.	89015	–
num. mol.	67826	20325
min. ΔG_{solv}, kJ/mol	$(-9.483 \pm 0.007) \cdot 10^1$	$(-5.636 \pm 0.037) \cdot 10^1$
min. ΔG_{solv} SMILES	NC1=NC(=O)N=C(N)N1	NC(=O)CCNC(N)=O
max. RMSE(ΔG_{solv}), kJ/mol	1.807	$6.589 \cdot 10^{-1}$
max. RMSE(ΔG_{solv}) SMILES	N=C1NC(N)=C(C=O)O1	C1C2OC1C21CC1
STD ΔG_{solv}, kJ/mol	9.977	7.100
max. D, debye	$(1.338 \pm 0.000) \cdot 10^1$	8.532 ± 0.041
max. D SMILES	CN1C=NC(=O)N=C1N	O=C1NCCNC(=O)N1
max. RMSE(D), debye	$9.450 \cdot 10^{-1}$	$3.325 \cdot 10^{-1}$
max. RMSE(D) SMILES	N=C1OCC=C1NC=O	C1C2CN3C1C1OC1C23
STD D, debye	1.831	1.355
max. RMSE($\Delta\epsilon$), eV	1.067	1.067
max. RMSE($\Delta\epsilon$) SMILES	CCC1CC12C1CC2C1	CCC1CC12C1CC2C1
	EGP and EGP* intersection	
tot. num. mol.	9437	–
num. mol.	6190	863
min. ΔG_{solv}, kJ/mol	$(-9.474 \pm 0.006) \cdot 10^1$	$(-7.814 \pm 0.019) \cdot 10^1$
min. ΔG_{solv} SMILES	NC1=NC(=O)N=C(N)N1	NS(=O)(=O)CCCCCS(N)(=O)=O
max. RMSE(ΔG_{solv}), kJ/mol	$6.960 \cdot 10^{-1}$	$3.500 \cdot 10^{-1}$
max. RMSE(ΔG_{solv}) SMILES	N=C1NC=CN1C(N)=O	ClCC1CCCCO1
STD ΔG_{solv}, kJ/mol	9.320	8.267
max. D, debye	$(1.326 \pm 0.000) \cdot 10^1$	8.050 ± 0.048
max. D SMILES	NC1=NC(=O)N=C(N)N1	O=S(=O)(F)CCS(=O)(=O)F
max. RMSE(D), debye	$6.943 \cdot 10^{-1}$	$3.179 \cdot 10^{-1}$
max. RMSE(D) SMILES	N=CNC1=NC=C(N)O1	N#CC1CCC(C#N)CC1
STD D, debye	1.881	1.492
max. RMSE($\Delta\epsilon$), eV	$2.916 \cdot 10^{-1}$	$2.916 \cdot 10^{-1}$
max. RMSE($\Delta\epsilon$) SMILES	C=C1CC1	C=C1CC1

decreased thermodynamic stability.

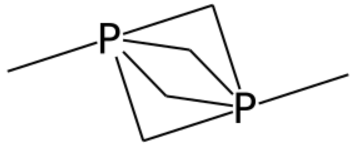

Figure S4: An EGP* molecule that cannot be transformed to or from methane through EGP* molecules using elementary mutations alone.

3 Additional data obtained during optimization in QM9* and EGP*

Full information about QM9* candidates, including the corresponding values of optimized quantities and how often they were proposed by our simulations, is presented in Table S3; we see that for all optimization problems but for maximizing D with strong $\Delta\epsilon$ constraint the agreement between simulations in terms of proposed candidates is close to unanimous. Figure S5 presents QM9* candidates that were not plotted in Figure 5; while we once again see MOSAiCS' ability to generate unconventional molecular structures, candidates C_7^{QM9}, C_8^{QM9}, and C_9^{QM9} demonstrate that our criterion for considering a molecule stable was not always realistic. To compare how fast MOSAiCS found candidates for different optimization problems and α_b values, we also calculated numbers of chemical graphs considered during a simulation before the candidate was encountered $N_{\mathrm{req}}^{\mathrm{graph}}$, total number of chemical graphs considered during a simulation $N_{\mathrm{tot}}^{\mathrm{graph}}$, and the number of global steps (as defined in Subsec. 2.5 of the main text) taken by a simulation before the candidate was encountered $N_{\mathrm{req}}^{\mathrm{step}}$. For QM9* simulations mean and standard deviation values of these quantities are summarized in Table S4. Changing the bias proportionality coefficient α_b did not significantly affect the number of global steps required on average to find a candidate molecule, but did lead to

a more thorough (but also costly) exploration of chemical space, as demonstrated by significant increase of $\overline{N_{\mathrm{req}}^{\mathrm{graph}}}$ and $\overline{N_{\mathrm{tot}}^{\mathrm{graph}}}$. Another interesting observation is that for a given $\Delta\epsilon$ constraint $N_{\mathrm{req}}^{\mathrm{step}}$ values are significantly higher for optimization of D than optimization of ΔG_{solv}. This is likely due to ΔG_{solv} being easier to optimize since free energy of solvation can be accurately decomposed as the sum of scalar contributions from different functional groups in the molecule,[S11] while for the dipole moment such contributions would take vector form whose sum would depend on the groups' placement in Cartesian space. Also note that for all optimization problems but for maximizing D with a strong $\Delta\epsilon$ constraint the $N_{\mathrm{req}}^{\mathrm{step}}$ was typically significantly smaller than the simulation length, which is an indicator of the simulations being converged in these cases. Relative improvement progress plots for optimization problems not shown in the main text are presented in Figure S6; while they look largely analogous to Figure 6, our simulations look less converged for minimization under strong $\Delta\epsilon$ constraint than under weak $\Delta\epsilon$ constraint. Plots of densities of QM9* molecules analogous to Figure 7 are found in Figures S7-S9; analogously to Figure 7 we observe an increase in diversity of considered molecules largely achieved by increasing exploration of chemical space regions less valuable in terms of properties of interest.

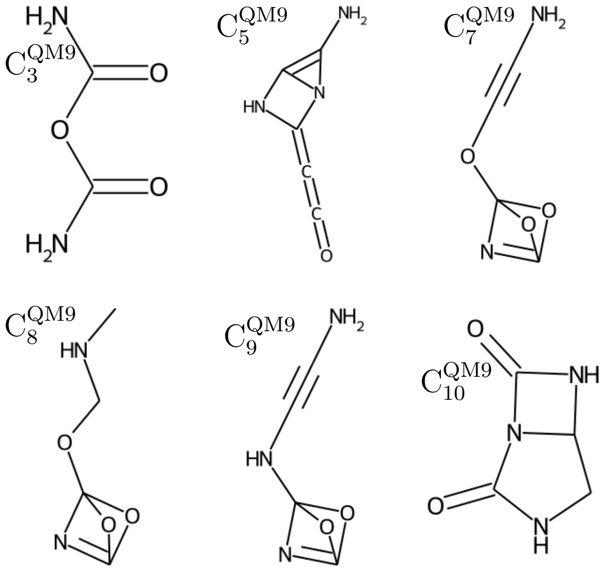

Figure S5: QM9* candidates that did not exhibit best target property values.

Table S3: QM9* candidates along with their corresponding optimized quantity values (opt. quant.) and the number of times the molecule was proposed as a candidate by simulations run with different values of the bias proportionality coefficient α_b (prop. with α_b). ΔG_{solv} and D values are in kJ/mol and debye.

molecule	SMILES	opt. quant.	prop. with α_b		
			0.0	0.2	0.4
min. ΔG_{solv} (weak $\Delta\epsilon$ constraint)					
C_1^{QM9}	NC1=NC(=O)N=C(N)N1	$(-9.479 \pm 0.006) \cdot 10^1$	8	8	8
min. ΔG_{solv} (strong $\Delta\epsilon$ constraint)					
C_2^{QM9}	NC(=O)NCNC(N)=O	$(-6.819 \pm 0.060) \cdot 10^1$	7	8	8
C_3^{QM9}	NC(=O)OC(N)=O	$(-6.727 \pm 0.000) \cdot 10^1$	1	0	0
max. D (weak $\Delta\epsilon$ constraint)					
C_4^{QM9}	NC1=C2OC(=CC=O)N12	$(1.573 \pm 0.010) \cdot 10^1$	8	7	8
C_5^{QM9}	NC1=C2NC(=C=C=O)N12	$(1.523 \pm 0.000) \cdot 10^1$	0	1	0
max. D (strong $\Delta\epsilon$ constraint)					
C_6^{QM9}	FC(F)=C=C=C1OCO1	$(1.114 \pm 0.000) \cdot 10^1$	1	4	1
C_7^{QM9}	NC#COC12N=C(O1)O2	$(1.083 \pm 0.003) \cdot 10^1$	4	2	2
C_8^{QM9}	CNCOC12N=C(O1)O2	$(1.051 \pm 0.020) \cdot 10^1$	0	1	4
C_9^{QM9}	NC#CNC12N=C(O1)O2	$(1.036 \pm 0.007) \cdot 10^1$	1	1	1
C_{10}^{QM9}	O=C1NCC2NC(=O)N12	$(1.000 \pm 0.000) \cdot 10^1$	2	0	0

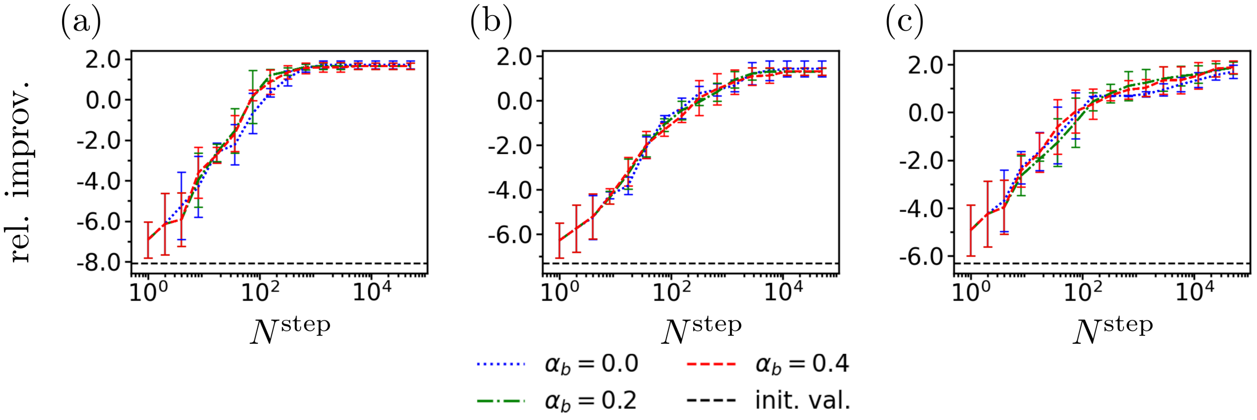

Figure S6: Relative improvement (as defined in Sec. 3 of the main text and estimated from $\Delta G_{\mathrm{solv}}^{\mathrm{cheap}}$ or D^{cheap}) observed at N^{step} global Monte Carlo steps for QM9* simulations optimizing (a) D with weak $\Delta\epsilon$ constraint, (b) ΔG_{solv} with strong $\Delta\epsilon$ constraint, and (c) D with strong $\Delta\epsilon$ constraint. For each value of the biasing potential α_b we plot the mean value over different random generator seeds, the error bars corresponding to standard deviation. "init. val." is the relative improvement of the simulations' starting molecule, *i.e.* methane.

Table S4: Mean and standard deviation of number of chemical graphs considered by a simulation before its candidate was encountered [$\overline{N_{\mathrm{req}}^{\mathrm{graph}}}$ and $\sigma(N_{\mathrm{req}}^{\mathrm{graph}})$], total number of chemical graphs considered by a simulation [$\overline{N_{\mathrm{tot}}^{\mathrm{graph}}}$ and $\sigma(N_{\mathrm{tot}}^{\mathrm{graph}})$], and number of global steps taken by a simulation before the candidate was encountered [$\overline{N_{\mathrm{req}}^{\mathrm{step}}}$ and $\sigma(N_{\mathrm{req}}^{\mathrm{step}})$] observed for trajectories in QM9* for different optimization problems (minimize free energy of solvation ΔG_{solv} or maximize dipole D under weak or strong constraint on HOMO-LUMO gap $\Delta\epsilon$) and different values of the bias proportionality coefficient α_b.

$\Delta\epsilon$ constr.	weak			strong		
α_b	0.0	0.2	0.4	0.0	0.2	0.4
			min. ΔG_{solv}			
$\overline{N_{\mathrm{req}}^{\mathrm{graph}}}$	$4.072 \cdot 10^3$	$5.164 \cdot 10^3$	$1.449 \cdot 10^4$	$3.854 \cdot 10^3$	$8.391 \cdot 10^3$	$8.544 \cdot 10^3$
$\sigma(N_{\mathrm{req}}^{\mathrm{graph}})$	$1.672 \cdot 10^3$	$2.979 \cdot 10^3$	$1.053 \cdot 10^4$	$2.347 \cdot 10^3$	$4.339 \cdot 10^3$	$6.707 \cdot 10^3$
$\overline{N_{\mathrm{tot}}^{\mathrm{graph}}}$	$2.352 \cdot 10^5$	$6.229 \cdot 10^5$	$6.862 \cdot 10^5$	$1.394 \cdot 10^5$	$4.304 \cdot 10^5$	$4.559 \cdot 10^5$
$\sigma(N_{\mathrm{tot}}^{\mathrm{graph}})$	$3.447 \cdot 10^3$	$1.588 \cdot 10^3$	$2.171 \cdot 10^3$	$3.188 \cdot 10^3$	$1.395 \cdot 10^3$	$1.677 \cdot 10^3$
$\overline{N_{\mathrm{req}}^{\mathrm{step}}}$	$4.234 \cdot 10^2$	$3.231 \cdot 10^2$	$7.781 \cdot 10^2$	$6.704 \cdot 10^2$	$5.584 \cdot 10^2$	$5.181 \cdot 10^2$
$\sigma(N_{\mathrm{req}}^{\mathrm{step}})$	$1.786 \cdot 10^2$	$1.823 \cdot 10^2$	$5.627 \cdot 10^2$	$3.729 \cdot 10^2$	$2.785 \cdot 10^2$	$4.025 \cdot 10^2$
			max. D			
$\overline{N_{\mathrm{req}}^{\mathrm{graph}}}$	$2.126 \cdot 10^4$	$3.131 \cdot 10^4$	$5.182 \cdot 10^4$	$1.164 \cdot 10^5$	$2.290 \cdot 10^5$	$2.181 \cdot 10^5$
$\sigma(N_{\mathrm{req}}^{\mathrm{graph}})$	$1.589 \cdot 10^4$	$1.762 \cdot 10^4$	$4.687 \cdot 10^4$	$3.757 \cdot 10^4$	$1.424 \cdot 10^5$	$1.285 \cdot 10^5$
$\overline{N_{\mathrm{tot}}^{\mathrm{graph}}}$	$2.426 \cdot 10^5$	$6.591 \cdot 10^5$	$7.169 \cdot 10^5$	$2.022 \cdot 10^5$	$4.765 \cdot 10^5$	$4.842 \cdot 10^5$
$\sigma(N_{\mathrm{tot}}^{\mathrm{graph}})$	$4.052 \cdot 10^3$	$9.418 \cdot 10^2$	$1.312 \cdot 10^3$	$7.042 \cdot 10^3$	$1.593 \cdot 10^3$	$1.150 \cdot 10^3$
$\overline{N_{\mathrm{req}}^{\mathrm{step}}}$	$2.779 \cdot 10^3$	$1.896 \cdot 10^3$	$2.886 \cdot 10^3$	$2.482 \cdot 10^4$	$2.063 \cdot 10^4$	$1.851 \cdot 10^4$
$\sigma(N_{\mathrm{req}}^{\mathrm{step}})$	$2.357 \cdot 10^3$	$1.099 \cdot 10^3$	$2.661 \cdot 10^3$	$9.271 \cdot 10^3$	$1.474 \cdot 10^4$	$1.314 \cdot 10^4$

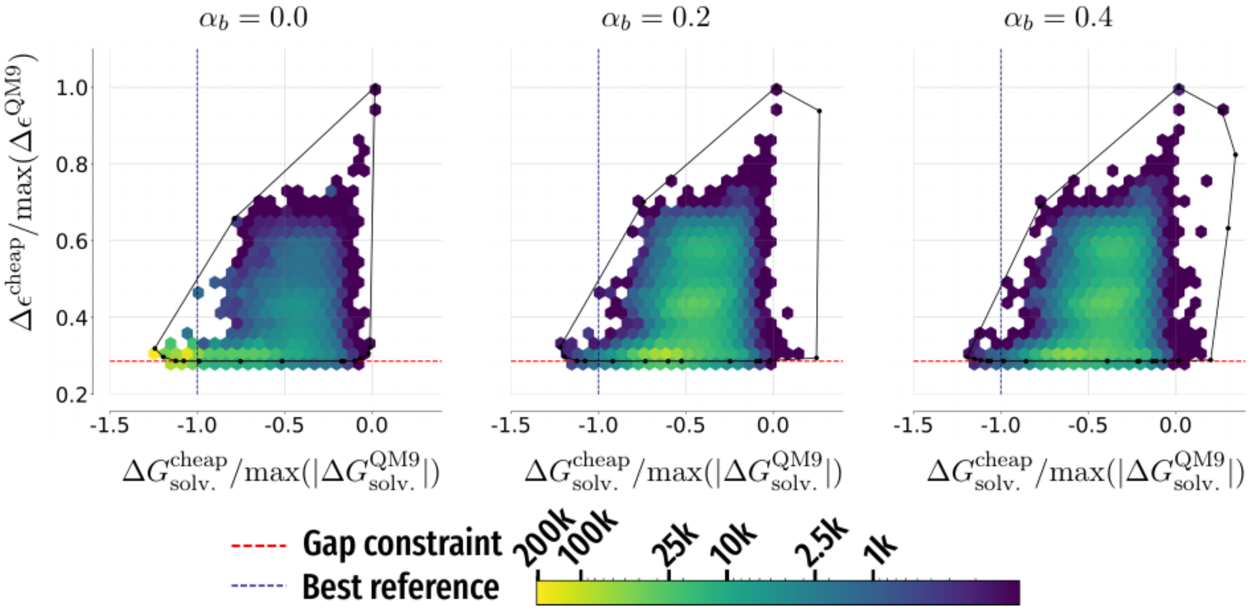

Figure S7: Densities of molecules encountered by example simulations minimizing free energy of solvation ΔG_{solv} with strong HOMO-LUMO gap $\Delta\epsilon$ constraint in QM9* that produced the candidate with smallest ΔG_{solv} for a given value of bias proportionality coefficient α_b.

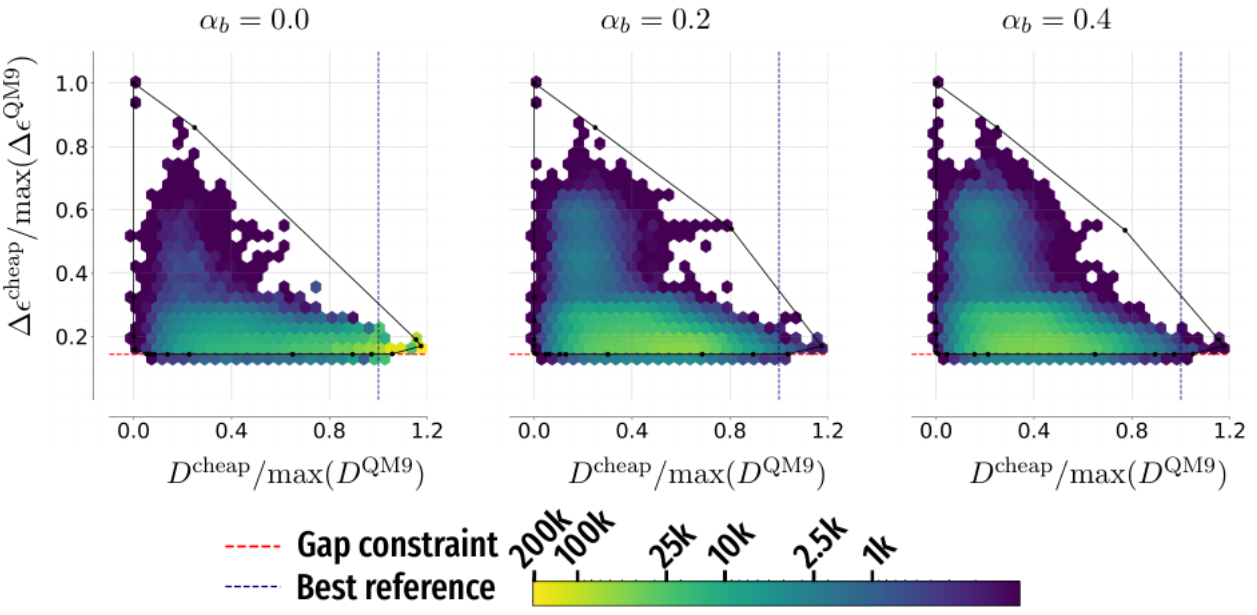

Figure S8: Densities of molecules encountered by example simulations maximizing dipole D with weak HOMO-LUMO gap $\Delta\epsilon$ constraint in QM9* that produced the candidate with largest D for a given value of bias proportionality coefficient α_b.

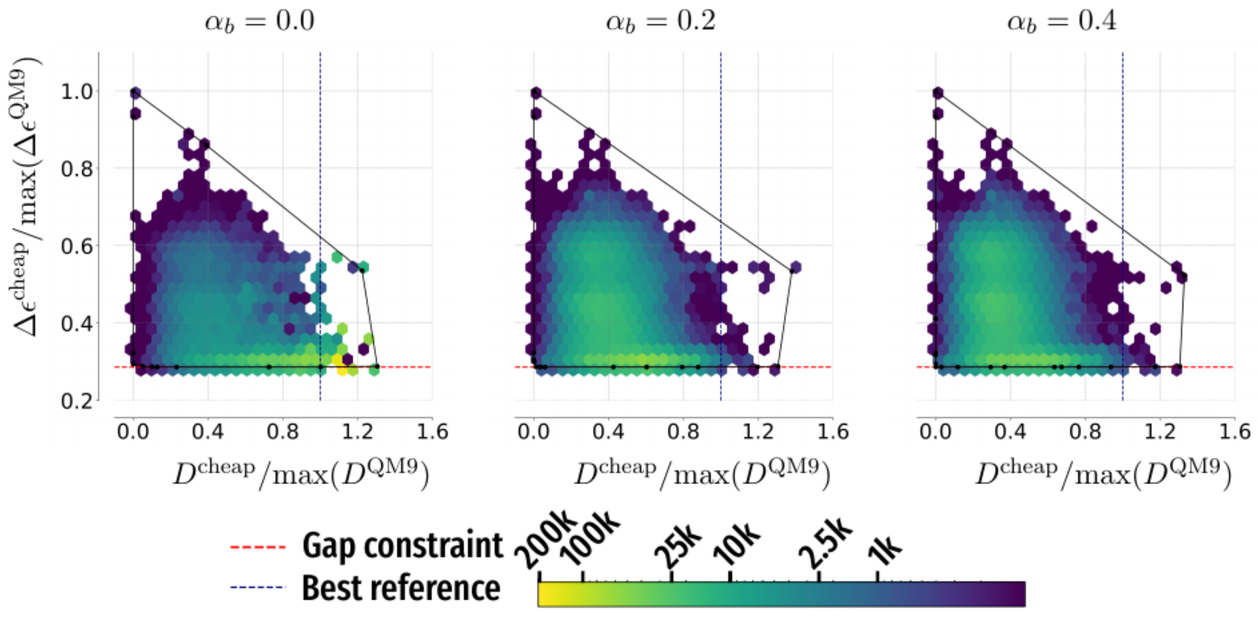

Figure S9: Densities of molecules encountered by example simulations maximizing dipole D with strong HOMO-LUMO gap $\Delta\epsilon$ constraint in QM9* that produced the candidate with largest D for a given value of bias proportionality coefficient α_b.

Due to the large number of candidates found in EGP* their full lists are left for Subsec. 3.2. We summarize how well these molecules were optimized for different optimization problems and α_b values in Table S5; we observe from the behavior of average optimized property values of candidates $\overline{D^{\text{best}}}$ and $\overline{\Delta G^{\text{best}}_{\text{solv}}}$ that adding biasing potential at worst did not significantly affect optimization results and at best provided significant improvements. Table S5 summarizes the behavior of $N^{\text{graph}}_{\text{req}}$, $N^{\text{graph}}_{\text{tot}}$, and $N^{\text{step}}_{\text{req}}$ for our EGP* simulations. Increasing α_b consistently increased the number of molecules explored by a trajectory; the $N^{\text{step}}_{\text{req}}$ values comparable to the total simulation length is an indicator of these simulations not being converged. Analogously to Figure 9, Figure S10 shows how using non-zero α_b could accelerate finding better candidates w.r.t the number of global Monte Carlo steps. However, we need to underline that, as indicated by $N^{\text{graph}}_{\text{req}}$ and $N^{\text{graph}}_{\text{tot}}$ values in Table S5, a non-zero α_b actually increased the computational time spent on proposing a candidate since MOSAiCS considered more chemical graphs during the corresponding simulations. Plots of densities of EGP* molecules analogous to Figure 10 are found in Figures S11-S13; analogously to Figure 10 we observe that increasing α_b tended to help the simulations explore

regions of chemical space more valuable in terms of optimized quantities.

Table S5: Numbers of chemical graphs considered by a trajectory before the candidate molecule was encountered $N_{\mathrm{req}}^{\mathrm{graph}}$, total numbers of chemical graphs considered by a trajectory $N_{\mathrm{tot}}^{\mathrm{graph}}$, and numbers of global steps after which the candidate was encountered [$\overline{N_{\mathrm{req}}^{\mathrm{step}}}$ and $\sigma(N_{\mathrm{req}}^{\mathrm{step}})$] observed for EGP* simulations; results are labeled analogously to Table S4.

$\Delta\epsilon$ constr.	weak			strong		
α_b	0.0	0.2	0.4	0.0	0.2	0.4
			min. ΔG_{solv}			
$\overline{N_{\mathrm{req}}^{\mathrm{graph}}}$	$1.533 \cdot 10^5$	$5.509 \cdot 10^5$	$4.916 \cdot 10^5$	$2.518 \cdot 10^5$	$4.992 \cdot 10^5$	$4.442 \cdot 10^5$
$\sigma(N_{\mathrm{req}}^{\mathrm{graph}})$	$3.857 \cdot 10^4$	$1.428 \cdot 10^5$	$1.658 \cdot 10^5$	$3.899 \cdot 10^4$	$2.052 \cdot 10^5$	$2.479 \cdot 10^5$
$\overline{N_{\mathrm{tot}}^{\mathrm{graph}}}$	$1.824 \cdot 10^5$	$6.895 \cdot 10^5$	$7.736 \cdot 10^5$	$3.085 \cdot 10^5$	$7.555 \cdot 10^5$	$8.969 \cdot 10^5$
$\sigma(N_{\mathrm{tot}}^{\mathrm{graph}})$	$1.236 \cdot 10^4$	$8.907 \cdot 10^3$	$6.908 \cdot 10^3$	$1.047 \cdot 10^4$	$6.802 \cdot 10^3$	$4.660 \cdot 10^3$
$\overline{N_{\mathrm{req}}^{\mathrm{step}}}$	$3.841 \cdot 10^4$	$3.963 \cdot 10^4$	$3.119 \cdot 10^4$	$3.777 \cdot 10^4$	$3.228 \cdot 10^4$	$2.428 \cdot 10^4$
$\sigma(N_{\mathrm{req}}^{\mathrm{step}})$	$1.174 \cdot 10^4$	$1.091 \cdot 10^4$	$1.095 \cdot 10^4$	$7.706 \cdot 10^3$	$1.406 \cdot 10^4$	$1.399 \cdot 10^4$
			max. D			
$\overline{N_{\mathrm{req}}^{\mathrm{graph}}}$	$2.141 \cdot 10^5$	$5.646 \cdot 10^5$	$6.574 \cdot 10^5$	$2.759 \cdot 10^5$	$5.532 \cdot 10^5$	$5.779 \cdot 10^5$
$\sigma(N_{\mathrm{req}}^{\mathrm{graph}})$	$2.881 \cdot 10^4$	$1.192 \cdot 10^5$	$7.904 \cdot 10^4$	$3.755 \cdot 10^4$	$1.794 \cdot 10^5$	$2.566 \cdot 10^5$
$\overline{N_{\mathrm{tot}}^{\mathrm{graph}}}$	$2.537 \cdot 10^5$	$7.740 \cdot 10^5$	$8.427 \cdot 10^5$	$3.269 \cdot 10^5$	$8.604 \cdot 10^5$	$9.540 \cdot 10^5$
$\sigma(N_{\mathrm{tot}}^{\mathrm{graph}})$	$1.296 \cdot 10^4$	$1.401 \cdot 10^4$	$1.016 \cdot 10^4$	$1.532 \cdot 10^4$	$7.360 \cdot 10^3$	$2.848 \cdot 10^3$
$\overline{N_{\mathrm{req}}^{\mathrm{step}}}$	$3.905 \cdot 10^4$	$3.617 \cdot 10^4$	$3.882 \cdot 10^4$	$4.033 \cdot 10^4$	$3.150 \cdot 10^4$	$3.001 \cdot 10^4$
$\sigma(N_{\mathrm{req}}^{\mathrm{step}})$	$6.732 \cdot 10^3$	$8.028 \cdot 10^3$	$4.668 \cdot 10^3$	$5.221 \cdot 10^3$	$1.073 \cdot 10^4$	$1.360 \cdot 10^4$

Lastly, the difference between cheap and converged estimates of D and ΔG_{solv} is very small for rigid molecules that only have one local minimum in configuration space (*e.g.* benzene), but it can become significant for larger non-rigid molecules with many valid conformers. To compare how much this difference affected candidate molecules obtained in this work we introduced "numerical error measure" $\Delta_{\mathrm{cheap}}^{\mathrm{conv}}$ defined as the ratio of mean absolute error of the cheap estimate relative to the converged one divided by standard deviation of the converged estimate over the pre-final (as defined in Subsec. 2.5 of the main text) molecules. Its values for QM9* and EGP* simulations are presented in Table S6. For QM9*, we note that maximizing D under strong $\Delta\epsilon$ constraint was apparently much more affected by using D^{cheap} in the minimization function, which may be the reason simulations for that optimiza-

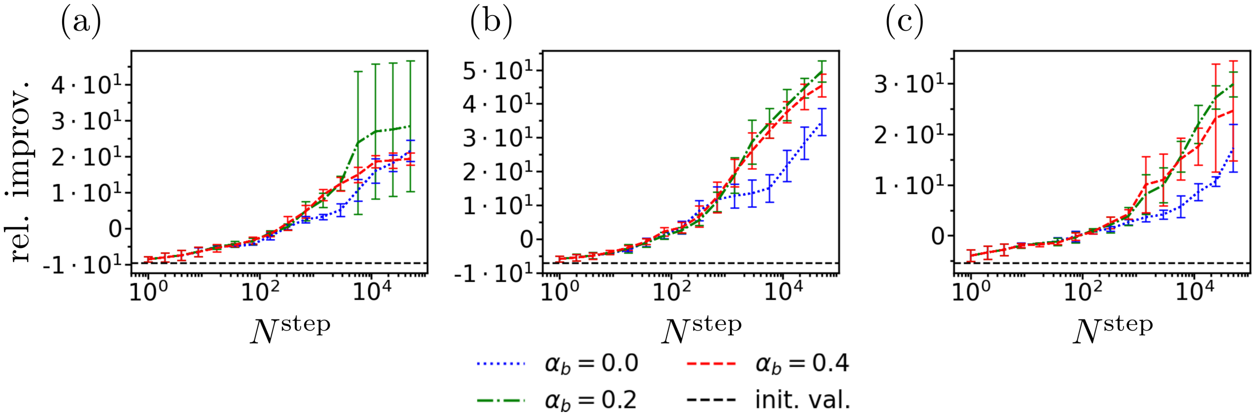

Figure S10: Relative improvement observed at N^{step} global Monte Carlo steps for EGP* simulations optimizing (a) D with weak $\Delta\epsilon$ constraint, (b) ΔG_{solv} with strong $\Delta\epsilon$ constraint, and (c) D with strong $\Delta\epsilon$ constraint. Results are organized analogously to Figure S6.

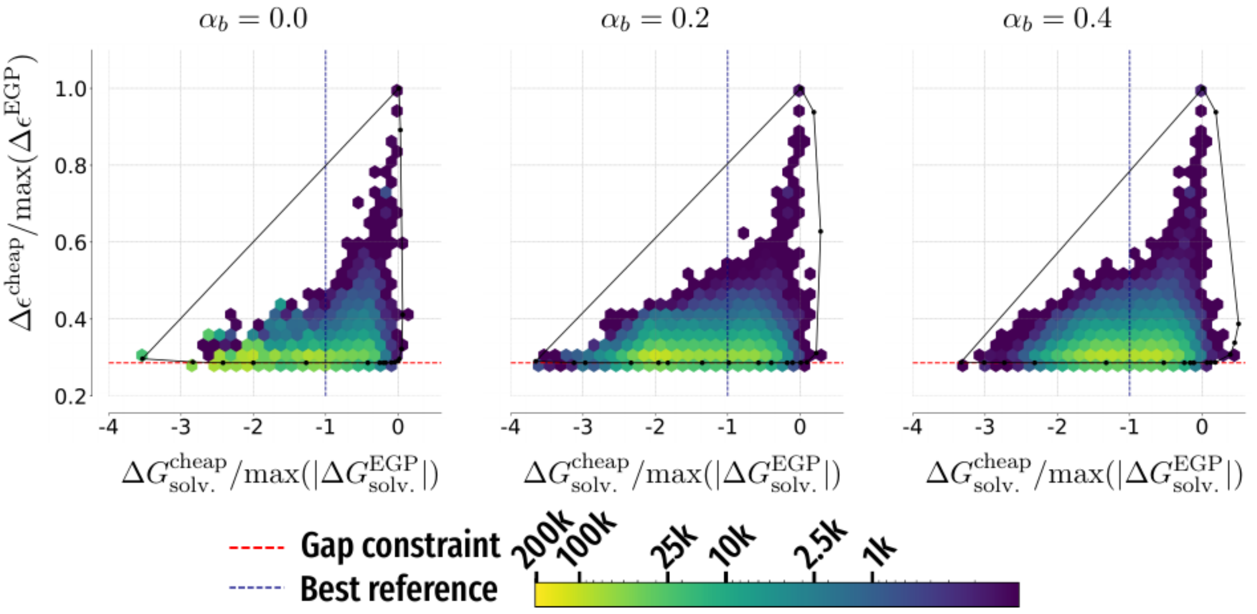

Figure S11: Densities of molecules encountered by simulations minimizing free energy of solvation ΔG_{solv} with strong HOMO-LUMO gap $\Delta\epsilon$ constraint in EGP* that produced the candidate with smallest ΔG_{solv} for a given value of bias proportionality coefficient α_b.

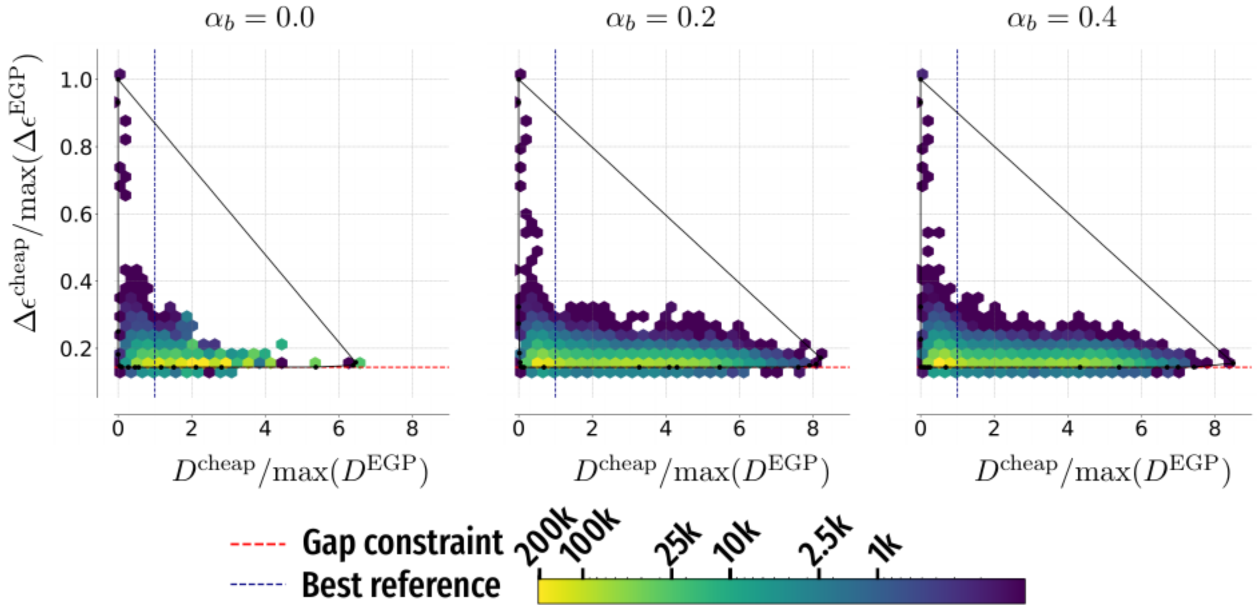

Figure S12: Densities of molecules encountered by simulations maximizing dipole D with weak HOMO-LUMO gap $\Delta\epsilon$ constraint in EGP* that produced the candidate with largest D for a given value of bias proportionality coefficient α_b.

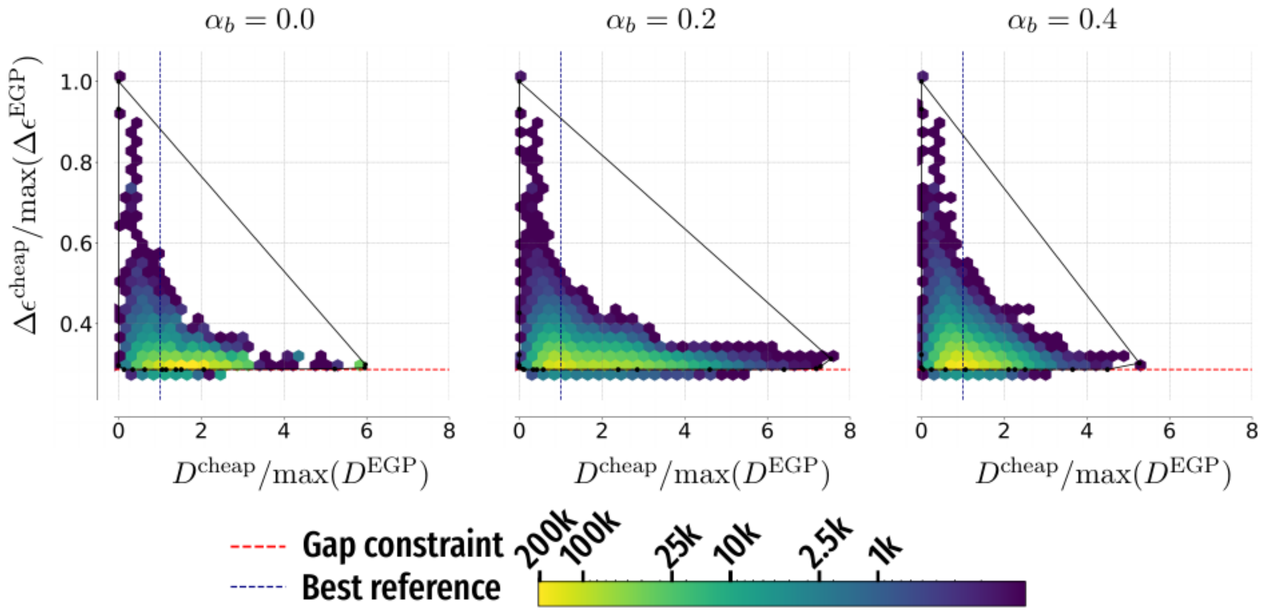

Figure S13: Densities of molecules encountered by simulations maximizing dipole D with strong HOMO-LUMO gap $\Delta\epsilon$ constraint in EGP* that produced the candidate with largest D for a given value of bias proportionality coefficient α_b.

tion problem agreed on candidate molecules much less frequently than for the other three. We also observe that for a given optimization problem simulations in EGP* were much more affected by cheap/converged difference than the ones in QM9*, likely due to EGP* consisting of larger molecules with more conformers to be accounted for. This added to EGP* being the more challenging set of molecules to search through compared to QM9*.

Table S6: Measure of error caused by using cheap version of optimized quantity during the simulation $\Delta^{\mathrm{conv}}_{\mathrm{cheap}}$ (see Sec. 3 for definition) observed during simulations in QM9* or EGP*, while maximizing dipole D or minimizing free energy of solvation ΔG_{solv}, under a weak or strong constraint on the HOMO-LUMO gap $\Delta\epsilon$, and with different values of the bias proportionality coefficient α_b.

Optimization problem	$\Delta^{\mathrm{conv}}_{\mathrm{cheap}}$		
	$\alpha_b = 0.0$	$\alpha_b = 0.2$	$\alpha_b = 0.4$
QM9*			
min. ΔG_{solv} (weak $\Delta\epsilon$ constraint)	$2.009 \cdot 10^{-1}$	$2.013 \cdot 10^{-1}$	$2.010 \cdot 10^{-1}$
min. ΔG_{solv} (strong $\Delta\epsilon$ constraint)	$1.827 \cdot 10^{-1}$	$1.814 \cdot 10^{-1}$	$1.834 \cdot 10^{-1}$
max. D (weak $\Delta\epsilon$ constraint)	$2.635 \cdot 10^{-1}$	$2.658 \cdot 10^{-1}$	$2.714 \cdot 10^{-1}$
max. D (strong $\Delta\epsilon$ constraint)	$4.108 \cdot 10^{-1}$	$4.189 \cdot 10^{-1}$	$4.270 \cdot 10^{-1}$
EGP*			
min. ΔG_{solv} (weak $\Delta\epsilon$ constraint)	$2.889 \cdot 10^{-1}$	$8.493 \cdot 10^{-1}$	$7.993 \cdot 10^{-1}$
min. ΔG_{solv} (strong $\Delta\epsilon$ constraint)	$4.145 \cdot 10^{-1}$	$5.359 \cdot 10^{-1}$	$4.409 \cdot 10^{-1}$
max. D (weak $\Delta\epsilon$ constraint)	$5.687 \cdot 10^{-1}$	$8.752 \cdot 10^{-1}$	$9.565 \cdot 10^{-1}$
max. D (strong $\Delta\epsilon$ constraint)	$4.722 \cdot 10^{-1}$	$8.612 \cdot 10^{-1}$	$7.068 \cdot 10^{-1}$

3.1 Effect of initial replica positions on optimization results

It is natural to assume that increasing diversity of molecules initially occupied by replicas should make it easier for MOSAiCS to propose better candidate molecules. We tested this hypothesis on minimizing ΔG_{solv} with strong $\Delta\epsilon$ constraint over QM9*, since it is a problem for which converged results proved easy to obtain and for which improvement over QM9 was observed. For that optimization problem we launched 8 simulations with $\alpha_b = 0.0$ which only differed from the others presented in this work by the choice of initial molecules: each was chosen with uniform probability from molecules in the intersection of QM9 and QM9* that

satisfied strong constraint on $\Delta\epsilon^{\mathrm{cheap}}$, then initial molecules were assigned to replicas in such a way that molecules with larger $\Delta G_{\mathrm{solv}}^{\mathrm{cheap}}$ were occupied by replicas with smaller $\beta^{(i)}$. The resulting mean optimization progress is plotted in Figure S14, with the corresponding values observed when all replicas were initialized in methane added for comparison. Surprisingly, while simulations with random starting molecules had significantly larger starting values of relative improvement, they failed to go beyond those values significantly earlier than simulations initialized with methane, and they all proposed previously mentioned $\mathrm{C}_2^{\mathrm{QM9}}$ as the candidate. While there might be better ways to choose initial molecules that would make search for candidates tangibly faster, finding them was beyond the scope of this work.

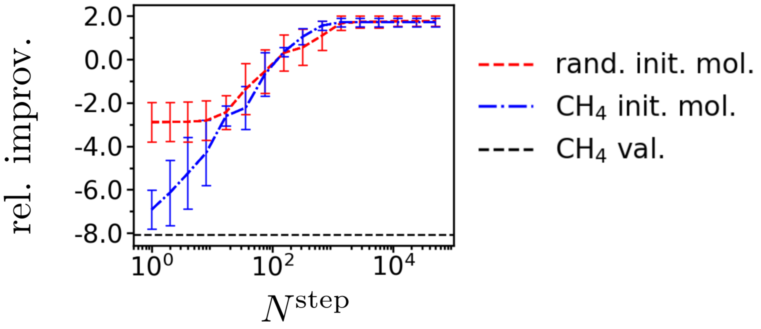

Figure S14: Relative improvement observed at N^{step} global Monte Carlo steps for QM9* simulations optimizing ΔG_{solv} with strong $\Delta\epsilon$ constraint if the molecules initially occupied by replicas are chosen randomly from QM9 dataset ("rand. init. mol.") with bias proportionality coefficient α_b set to 0.0. Plotted for comparison are corresponding values when all replicas are initialized at methane ("CH_4 init. mol.", also appearing in Figure S6a) as well as the relative improvement value for methane ("CH_4 val.").

3.2 EGP* full candidate list

EGP* candidates proposed by MOSAiCS are listed in Tables S7-S10. Examples of candidates plotted in Figure S15 demonstrate MOSAiCS' ability to produce candidates with complicated structures, including unusual heterocycles ($\mathrm{C}_{14}^{\mathrm{EGP}}$, $\mathrm{C}_{38}^{\mathrm{EGP}}$, and $\mathrm{C}_{82}^{\mathrm{EGP}}$), conjugated heterocycles ($\mathrm{C}_{17}^{\mathrm{EGP}}$, $\mathrm{C}_{40}^{\mathrm{EGP}}$), and caged molecules ($\mathrm{C}_{32}^{\mathrm{EGP}}$).

Table S7: EGP* candidates proposed during minimization of free energy of solvation ΔG_{solv} with weak constraint on the HOMO-LUMO gap $\Delta\epsilon$, the corresponding values of ΔG_{solv}, and the number of times a molecule was proposed as a candidate by simulations run with different values of the bias proportionality coefficient α_b (prop. with α_b).

molecule	SMILES	ΔG_{solv}, kJ/mol	prop. with α_b		
			0.0	0.2	0.4
C_1^{EGP}	CS(C)=P(C)(C)[Si](C)(Cl)S[Si]1=P[Si]1(Br)Br	$(-1.194 \pm 0.007) \cdot 10^3$	0	0	1
C_2^{EGP}	CS(C)(C)CCP1CS1=P(=O)SP(=O)=O	$(-1.080 \pm 0.001) \cdot 10^3$	0	1	2
C_3^{EGP}	CS(C)(C)CNP1CS1=P(=O)SP(=O)=O	$(-1.041 \pm 0.001) \cdot 10^3$	0	1	0
C_4^{EGP}	CS(C)(C)CC(Br)S[Si](Br)(Br)SP(=O)=O	$(-9.896 \pm 0.092) \cdot 10^2$	0	2	1
C_5^{EGP}	CS(C)(C)CNP1NS1=P(=O)SP(=O)=O	$(-9.868 \pm 0.050) \cdot 10^2$	0	0	1
C_6^{EGP}	CS(C)(C)CS[Si]1(Br)S[Si]1(Br)SP(=O)=O	$(-9.660 \pm 0.075) \cdot 10^2$	0	0	1
C_7^{EGP}	CS(C)(C)CP(Br)S[Si](Br)(Br)SP(=O)=O	$(-9.573 \pm 0.127) \cdot 10^2$	0	1	0
C_8^{EGP}	CS1(C)CCS(=P(=O)SP(=O)=O)P(Cl)C1	$(-9.556 \pm 0.055) \cdot 10^2$	0	1	0
C_9^{EGP}	CS(C)(C)CC(Cl)S[Si](Br)(Br)SP(=O)=O	$(-9.405 \pm 0.226) \cdot 10^2$	0	0	1
C_{10}^{EGP}	CS1(C)CCP(Cl)[Si](Br)(SP(=O)=O)SC1	$(-9.244 \pm 0.092) \cdot 10^2$	0	1	0
C_{11}^{EGP}	CS(=NP(=O)=S=P(=O)Br)NCS(C)(C)C	$(-8.418 \pm 0.102) \cdot 10^2$	0	1	0
C_{12}^{EGP}	CS(P1CSP(SP(=O)=O)S1)=P(C)(C)N	$(-7.724 \pm 0.026) \cdot 10^2$	0	0	1
C_{13}^{EGP}	CS(C)(C)CCSNSP(=O)=CP(#N)Br	$(-7.144 \pm 0.056) \cdot 10^2$	1	0	0
C_{14}^{EGP}	CP1(=CN)CP(C)(N)=[Si](SP(=O)=O)S1	$(-5.121 \pm 0.076) \cdot 10^2$	1	0	0
C_{15}^{EGP}	CP1(N)=CP(SP(=O)=O)C(N)=P(C)(C)C1	$(-4.511 \pm 0.059) \cdot 10^2$	1	0	0
C_{16}^{EGP}	CP(C)(N)=C(N)NCOC(=N)SP(=O)=O	$(-4.456 \pm 0.079) \cdot 10^2$	1	0	0
C_{17}^{EGP}	CS(C)(C)N=CP1(N)=NP2(=NP(=O)=N2)O1	$(-4.261 \pm 0.003) \cdot 10^2$	1	0	0
C_{18}^{EGP}	CP(N)(N)=C1NCCOP(OP(=O)=O)N1	$(-4.153 \pm 0.006) \cdot 10^2$	1	0	0
C_{19}^{EGP}	CS(C)(=NCCS(#N)=NP(#N)Br)=C(N)N	$(-3.825 \pm 0.013) \cdot 10^2$	1	0	0
C_{20}^{EGP}	CP1(N)=C(N)P(OP(=O)=O)S(=O)CCC1	$(-3.822 \pm 0.012) \cdot 10^2$	1	0	0

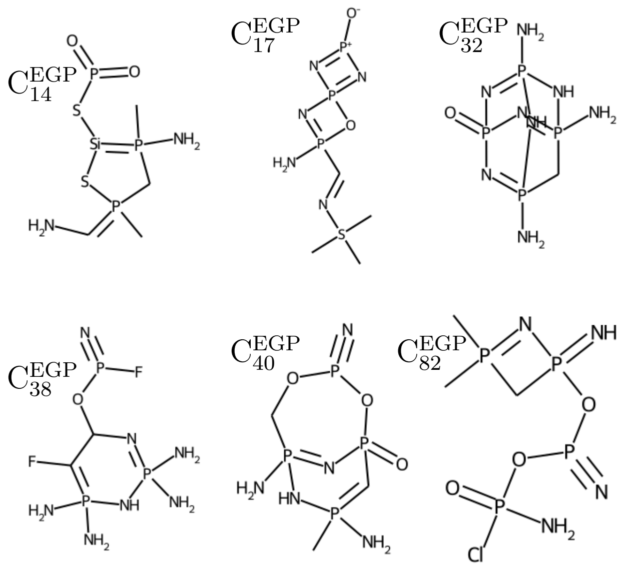

Figure S15: Examples of EGP* candidates.

Table S8: EGP* candidates proposed during minimization of free energy of solvation ΔG_{solv} with strong constraint on HOMO-LUMO gap $\Delta\epsilon$; columns are labeled analogously to Table S7.

molecule	SMILES	ΔG_{solv}, kJ/mol	prop. with α_b		
			0.0	0.2	0.4
C_{21}^{EGP}	COP(#N)OP(#N)NCCP(N)(N)=CN	$(-2.698 \pm 0.021) \cdot 10^2$	1	0	0
C_{22}^{EGP}	N#P(N)OP(#N)OCNCP(N)(N)=CN	$(-2.670 \pm 0.043) \cdot 10^2$	1	1	0
C_{23}^{EGP}	CC(OCOP(#N)OP(#N)N)=P(C)(C)N	$(-2.483 \pm 0.037) \cdot 10^2$	0	1	0
C_{24}^{EGP}	CP(N)(=CN)NCNP(#N)OP(#N)N	$(-2.480 \pm 0.014) \cdot 10^2$	0	1	0
C_{25}^{EGP}	N#P(F)OP(#N)NP1(=N)NC=P(N)(N)N1	$(-2.431 \pm 0.013) \cdot 10^2$	0	1	0
C_{26}^{EGP}	CC(CP(N)(N)=CN)NP(#N)OP(#N)N	$(-2.405 \pm 0.051) \cdot 10^2$	1	0	0
C_{27}^{EGP}	CP(C)(N)=COCCOP(#N)OP(#N)N	$(-2.383 \pm 0.042) \cdot 10^2$	1	0	0
C_{28}^{EGP}	N#P(F)OP(#N)OC1CC(=P(N)(N)N)O1	$(-2.376 \pm 0.007) \cdot 10^2$	0	1	0
C_{29}^{EGP}	COP(#N)NP(#N)NCCP(N)(N)=CN	$(-2.368 \pm 0.023) \cdot 10^2$	1	0	0
C_{30}^{EGP}	CP(C)(C)=COC(N)OP(#N)OP(#N)N	$(-2.366 \pm 0.007) \cdot 10^2$	0	0	1
C_{31}^{EGP}	CP(C)(N)=C(F)OCOP(#N)OP(#N)F	$(-2.335 \pm 0.020) \cdot 10^2$	0	1	0
C_{32}^{EGP}	NP12=NP3(=O)N=P(N)(C1)NP(N)(=N3)N2	$(-2.295 \pm 0.008) \cdot 10^2$	0	1	0
C_{33}^{EGP}	N#P(F)OP1(=O)C=P(N)(N)CP(N)(N)=N1	$(-2.291 \pm 0.010) \cdot 10^2$	0	1	0
C_{34}^{EGP}	CP1(N)=CP(=O)(OP(#N)N)N=P(N)(N)N1	$(-2.261 \pm 0.027) \cdot 10^2$	0	0	2
C_{35}^{EGP}	CP12=NP3(=O)N=P(N)(CP(N)(=N3)N1)N2	$(-2.257 \pm 0.007) \cdot 10^2$	0	0	1
C_{36}^{EGP}	NP12=NP3(=O)N=P(N)(N1)NP(N)(=N3)N2	$(-2.253 \pm 0.008) \cdot 10^2$	0	0	1
C_{37}^{EGP}	CP(N)(N)=C1COP(#N)OP(N)(=O)N1	$(-2.232 \pm 0.007) \cdot 10^2$	2	0	1
C_{38}^{EGP}	N#P(F)OC1N=P(N)(N)NP(N)(N)=C1F	$(-2.142 \pm 0.005) \cdot 10^2$	0	0	1
C_{39}^{EGP}	N#P(N)OP1(=O)C=P(N)(N)OP(N)(N)=N1	$(-2.099 \pm 0.019) \cdot 10^2$	0	0	1
C_{40}^{EGP}	CP1(N)=CP2(=O)N=P(N)(COP(#N)O2)N1	$(-2.076 \pm 0.009) \cdot 10^2$	1	0	0

Table S9: EGP* candidates proposed during maximization of dipole D with weak constraint on HOMO-LUMO gap $\Delta\epsilon$; columns are labeled analogously to Table S7.

molecule	SMILES	D, debye	prop. with α_b		
			0.0	0.2	0.4
C_{41}^{EGP}	CS(C)(C)CCC=PSP(#N)SP(=O)=O	$(1.095 \pm 0.009) \cdot 10^2$	0	1	0
C_{42}^{EGP}	CS(C)(C)CSC=CSP(#N)SP(=O)=O	$(1.075 \pm 0.009) \cdot 10^2$	0	1	0
C_{43}^{EGP}	O=P(=O)SP(=O)=S1N[Si]1=S1CP1CSCl	$(1.063 \pm 0.013) \cdot 10^2$	0	1	0
C_{44}^{EGP}	CP(SC=CSCS(C)(C)C)SP(=O)=S	$(1.029 \pm 0.010) \cdot 10^2$	0	0	1
C_{45}^{EGP}	CP(NCNCCS(C)(C)C)SP(=O)=O	$(1.012 \pm 0.010) \cdot 10^2$	0	1	0
C_{46}^{EGP}	CP(SC=CSCS(C)(C)C)SP(=O)=O	$(9.944 \pm 0.077) \cdot 10^1$	0	1	0
C_{47}^{EGP}	CS(C)(C)CCCCNP(F)SP(=O)=O	$(9.743 \pm 0.127) \cdot 10^1$	0	1	0
C_{48}^{EGP}	CS(C)(C)CCCS1=[Si](SP(=S)=S)C1N	$(9.713 \pm 0.073) \cdot 10^1$	0	0	1
C_{49}^{EGP}	CS(C)(C)CCNSNP(#N)SP(=O)=O	$(9.640 \pm 0.238) \cdot 10^1$	0	0	1
C_{50}^{EGP}	CS(C)(C)CCCCSP(F)SP(=O)=O	$(9.563 \pm 0.060) \cdot 10^1$	0	1	0
C_{51}^{EGP}	CS(C)(C)N=PSCSCSN=P(=O)Br	$(9.499 \pm 0.044) \cdot 10^1$	0	1	0
C_{52}^{EGP}	C=S(C)CSCP1CS1=P(=O)SP(=O)=O	$(9.420 \pm 0.082) \cdot 10^1$	0	0	1
C_{53}^{EGP}	CS(C)(C)NC=CSP(N=S)SP(=O)=O	$(9.267 \pm 0.026) \cdot 10^1$	0	0	1
C_{54}^{EGP}	CP(CCCSP(=O)=O)CCS(C)(C)C	$(9.264 \pm 0.110) \cdot 10^1$	0	0	1
C_{55}^{EGP}	CS(C)(N)C[Si]1(F)CSP(SP(=O)=S)S1	$(8.639 \pm 0.076) \cdot 10^1$	0	0	1
C_{56}^{EGP}	C=S(C)(C)=PSC1OP1SCSP(=O)=O	$(8.447 \pm 0.059) \cdot 10^1$	0	0	1
C_{57}^{EGP}	CS(=CN)CCNCSP(#N)SP(#N)Br	$(7.938 \pm 0.134) \cdot 10^1$	1	0	0
C_{58}^{EGP}	CS1(CSC=CSP(#N)SP(#N)Br)CO1	$(7.709 \pm 0.071) \cdot 10^1$	1	0	0
C_{59}^{EGP}	C=S(C)(C)=PC=CCSC(=O)SP(=O)=O	$(7.685 \pm 0.093) \cdot 10^1$	1	0	0
C_{60}^{EGP}	CS(C)(=CC=COS(=O)P(=O)=S)=C(N)N	$(7.587 \pm 0.020) \cdot 10^1$	1	0	0
C_{61}^{EGP}	C=S(C)(=CN)CCCSP(#N)SP(#N)Br	$(7.495 \pm 0.137) \cdot 10^1$	1	0	0
C_{62}^{EGP}	CP(C)(C)=C(N)NC=CCNCP(=S)=S	$(7.457 \pm 0.087) \cdot 10^1$	1	0	0
C_{63}^{EGP}	CP(C)(NC=CNC#CP(=S)=S)=C(N)N	$(7.453 \pm 0.009) \cdot 10^1$	1	0	0
C_{64}^{EGP}	CS(C)(=CN)=NSCC=CN=S=NC=O	$(5.369 \pm 0.012) \cdot 10^1$	1	0	0

Table S10: EGP* candidates proposed during maximization of dipole D with strong constraint on HOMO-LUMO gap $\Delta\epsilon$; columns are labeled analogously to Table S7.

molecule	SMILES	D, debye	prop. with α_b		
			0.0	0.2	0.4
C_{65}^{EGP}	NC=P(N)(N)CCCCNCN=S(=O)=O	$(5.981 \pm 0.114) \cdot 10^1$	0	1	0
C_{66}^{EGP}	CP(C)(C)=CCCCOP(#N)OP(#N)F	$(4.870 \pm 0.027) \cdot 10^1$	0	5	0
C_{67}^{EGP}	N#P(N)OP(#N)NCNCP(N)(N)=CN	$(4.865 \pm 0.073) \cdot 10^1$	0	1	0
C_{68}^{EGP}	C=P(C)(C)CCNCNP(#N)OP(#N)F	$(4.587 \pm 0.049) \cdot 10^1$	1	0	0
C_{69}^{EGP}	CP(C)(N)=C1CC(OP(#N)OP(#N)F)C1	$(4.401 \pm 0.084) \cdot 10^1$	0	1	0
C_{70}^{EGP}	C=P(C)(C)CCCOP(#N)OP(#N)F	$(4.024 \pm 0.038) \cdot 10^1$	1	0	0
C_{71}^{EGP}	C=P1(N)CC(OCOP(#N)OP(#N)F)C1	$(3.924 \pm 0.057) \cdot 10^1$	0	0	1
C_{72}^{EGP}	CC=P(C)(N)NCOP(#N)OP(#N)F	$(3.847 \pm 0.052) \cdot 10^1$	0	0	1
C_{73}^{EGP}	C=P(N)(N)C(N)CCNP(#N)OP(#N)F	$(3.842 \pm 0.099) \cdot 10^1$	0	0	1
C_{74}^{EGP}	C=P(C)(N)CNCOP(#N)OP(#N)F	$(3.777 \pm 0.035) \cdot 10^1$	0	0	2
C_{75}^{EGP}	C=P(C)(C)OCC(F)OP(#N)OP(#N)F	$(3.768 \pm 0.034) \cdot 10^1$	0	0	1
C_{76}^{EGP}	CP(N)(N)=CCCCCOCNP(#N)F	$(3.674 \pm 0.091) \cdot 10^1$	0	0	1
C_{77}^{EGP}	CC(CNP(=O)(F)NP(#N)F)=P(C)(C)N	$(3.427 \pm 0.070) \cdot 10^1$	0	0	1
C_{78}^{EGP}	CP(C)(N)=CCC(F)(F)CCOP(#N)F	$(3.195 \pm 0.048) \cdot 10^1$	1	0	0
C_{79}^{EGP}	C=P(C)(N)CCOP(#N)OCOP(#N)F	$(3.134 \pm 0.126) \cdot 10^1$	1	0	0
C_{80}^{EGP}	CC=P(C)(N)CC(F)CCOP(#N)F	$(2.977 \pm 0.052) \cdot 10^1$	1	0	0
C_{81}^{EGP}	N#P(F)NCCC12C=P(N)(NCN1)NC2	$(2.870 \pm 0.041) \cdot 10^1$	1	0	0
C_{82}^{EGP}	CP1(C)=NP(=N)(OP(#N)OP(N)(=O)Cl)C1	$(2.643 \pm 0.006) \cdot 10^1$	1	0	0
C_{83}^{EGP}	CP(C)(C)=N[Si]1(N)COC(OP(#N)F)C1	$(2.588 \pm 0.017) \cdot 10^1$	1	0	0

References

(S1) Ebejer, J.-P.; Morris, G. M.; Deane, C. M. Freely Available Conformer Generation Methods: How Good Are They? *J. Chem. Inf. Model.* *52*, 1146–1158.

(S2) https://kjelljorner.github.io/morfeus.

(S3) (a) Halgren, T. A. Merck molecular force field. I. Basis, form, scope, parameterization, and performance of MMFF94. *J. Comput. Chem.* **1996**, *17*, 490–519; (b) Halgren, T. A. Merck molecular force field. II. MMFF94 van der Waals and electrostatic parameters for intermolecular interactions. *J. Comput. Chem.* **1996**, *17*, 520–552; (c) Halgren, T. A. Merck molecular force field. III. Molecular geometries and vibrational frequencies for MMFF94. *J. Comput. Chem.* **1996**, *17*, 553–586; (d) Halgren, T. A.; Nachbar, R. B. Merck molecular force field. IV. Conformational energies and geometries for MMFF94. *J. Comput. Chem.* **1996**, *17*, 587–615; (e) Halgren, T. A. Merck molecular force field. V. Extension of MMFF94 using experimental data, additional computational data, and empirical rules. *J. Comput. Chem.* **1996**, *17*, 616–641; (f) Halgren, T. A. MMFF VI. MMFF94s option for energy minimization studies. *J. Comput. Chem.* **1999**, *20*, 720–729; (g) Halgren, T. A. MMFF VII. Characterization of MMFF94, MMFF94s, and other widely available force fields for conformational energies and for intermolecular-interaction energies and geometries. *J. Comput. Chem.* **1999**, *20*, 730–748.

(S4) Tosco, P.; Stiefl, N.; Landrum, G. Bringing the MMFF force field to the RDKit: implementation and validation. *J. Cheminform.* **2014**, *6*, 37.

(S5) RDKit: Open-source cheminformatics. https://www.rdkit.org.

(S6) Kim, Y.; Kim, W. Y. Universal Structure Conversion Method for Organic Molecules: From Atomic Connectivity to Three-Dimensional Geometry. *BKCS* **2015**, *36*, 1769–1777.

(S7) https://github.com/jensengroup/xyz2mol.

(S8) Bannwarth, C.; Caldeweyher, E.; Ehlert, S.; Hansen, A.; Pracht, P.; Seibert, J.; Spicher, S.; Grimme, S. Extended tight-binding quantum chemistry methods. *WIREs Computational Molecular Science* **2021**, *11*, e1493.

(S9) Bannwarth, C.; Ehlert, S.; Grimme, S. GFN2-xTB-An Accurate and Broadly Parametrized Self-Consistent Tight-Binding Quantum Chemical Method with Multipole Electrostatics and Density-Dependent Dispersion Contributions. *J. Chem. Theory Comput.* **2019**, *15*, 1652–1671.

(S10) Ehlert, S.; Stahn, M.; Spicher, S.; Grimme, S. Robust and Efficient Implicit Solvation Model for Fast Semiempirical Methods. *J. Chem. Theory Comput.* **2021**, *17*, 4250–4261.

(S11) Chung, Y.; Gillis, R. J.; Green, W. H. Temperature-dependent vapor–liquid equilibria and solvation free energy estimation from minimal data. *AIChE J.* **2020**, *66*, e16976.
